# Supplementary material for: A national survey of Russian physicians' knowledge of diagnosis and management of food-induced anaphylaxis
Source: BMJ Open. 2017 Jul 20;7(7):e015901. doi: 10.1136/bmjopen-2017-015901 (PMC5541513; doi:10.1136/bmjopen-2017-015901)
Supplement: Supplementary data [file bmjopen-2017-015901supp001.pdf]

Supplementary document 1

We would be very grateful for your participation in our study by completing this survey. It should take you approximately 10 minutes to complete. We will not collect any information that could identify you personally or the hospital where you work.

In this survey you will find three clinical scenarios and we would appreciate if you could address them according to your own clinical knowledge and expertise without seeking advice from other parties. It will help us to provide fair and objective evaluation of the results.

We hope to publish the results of the survey in 2016 and look forward to sharing these with you.

Sex:

- ☐ **Male**
- ☐ **Female**

Age: ___________

Country and city of residence:

Primary specialty:

Clinical practice settings:

A) primary care

B) primary practice

C) secondary care

D) tertiary care

Years in clinical practice:

☐ **1-2**

☐ **3-5**

☐ **6-10**

☐ **11-15**

☐ **More than 15**

Years in specialty practice (if applicable) ___________

Next page of the online questionnaire…

Clinical scenario 1:

During a busy weekend A&E shift, you are seeing a 6-year-old boy. His main complaint at admission is syncope. 10 minutes after eating Chinese meal at a friend's house he developed an urticarial rash on the neck. 5 minutes after the rash appearance he complained of abdominal cramping and vomited twice. After 15 minutes, he felt dizzy, developed a wheeze and fainted. He have been delivered to the hospital 35 minutes after occurrence of the first symptopms. It is not known if he has food allergy but he had 2 hospitalizations for asthma in the last year.

On examination, his heart rate is 90, blood pressure 90/45mmHg, respiratory rate 22, and oxygen saturation 94%. His weight is 22 kg. He has diffuse wheezing and is in moderate respiratory distress. He was not carrying his salbutamol inhaler and hasn't received any medications yet.

What is the most likely diagnosis?

____

Is any treatment required?

☐ Yes
☐ No

if yes, please choose the right answer/s (can be more than one option)

1) Oxygen
2) Salbutamol inhaler
3) Antihistamine (Cetirizine) orally
4) Antihistamine (Chlorphenamine) orally
5) Immediately start first line treatment - Antihistamine (Chlorphenamine) IV
6) Call PICU
7) Immediately start first line treatment - Adrenaline IM 1:10 000
8) Immediately start first line treatment - Adrenaline IM 1:1000
9) Metoclopramide
10) Adrenaline IV 1:100 if other treatment is unsuccessful
11) Immediately start first line treatment - Activated Charcoal
12) Inhaled Steroids
13) Oral prednisolone, in addition to first line treatment
14) Prednisolone IM as a first line treatment
15) Valproates
16) Midodrine
17) None of the above

if none of the above or any additional medications, please provide your options:

____

Next page of the online questionnaire…

On further questioning when the boy had recovered, he reported that the Chinese meal contained egg fried rice, beef, bean shoots, sesame, cashew nuts and peanuts. He has previously sometimes complained of itchy throat after eating chocolate and he had eczema in infancy worsened when eating egg. However, he has recently had egg without any reaction.

What tests would you do?

a) Full blood count
b) ECG
c) Echocardiogram
d) Urinalysis
e) Serum tryptase
f) Chest X-ray
g) EEG
h) sIgE or skin prick test to Egg
i) sIgE or skin prick test to Peanut, Cashew and Sesame
j) sIgG test to multiple food allergens
k) MRI
l) Spirometry
m) None of the above

if none of the above, or any additional tests, please provide your options:

____

____

What advice and further treatment would you give to this patient

____

____
