# Supplementary material for: A national survey of Russian physicians' knowledge of diagnosis and management of food-induced anaphylaxis
Source: BMJ Open. 2017 Jul 20;7(7):e015901. doi: 10.1136/bmjopen-2017-015901 (PMC5541513; doi:10.1136/bmjopen-2017-015901)
Supplement: Author's manuscript [file bmjopen-2017-015901.draft_revisions.pdf]

BMJ Open

To Russia with anaphylaxis: National survey of physicians knowledge of diagnosis and management of food induced anaphylaxis

Journal:	*BMJ Open*
Manuscript ID	bmjopen-2017-015901
Article Type:	Research
Date Submitted by the Author:	10-Jan-2017
Complete List of Authors:	Munblit, Daniel; Pervyj Moskovskij gosudarstvennyj medicinskij universitet imeni I M Secenova, Faculty of Pediatrics; Imperial College London, Paediatrics<br>Treneva, Marina; Veltischev Clinical Pediatric Research Institute of Pirogov Russian National Research Medical University; International Inflammation (in-FLAME) Network of the World Universities Network<br>Korsunskiy, Ilya; Pervyj Moskovskij gosudarstvennyj medicinskij universitet imeni I M Secenova, Faculty of Pediatrics<br>Asmanov, Alan; Veltischev Clinical Pediatric Research Institute of Pirogov Russian National Research Medical University<br>Pampura, Alexander; Veltischev Clinical Pediatric Research Institute of Pirogov Russian National Research Medical University; International Inflammation (in-FLAME) Network of the World Universities Network<br>Warner, John O.; Imperial College London, Paediatrics; International Inflammation (in-FLAME) Network of the World Universities Network
**Primary Subject Heading**:	Immunology (including allergy)
Secondary Subject Heading:	Paediatrics
Keywords:	anaphylaxis, survey, food-induced anaphylaxis, anaphylaxis knowledge

SCHOLARONE™
Manuscripts

For peer review only - http://bmjopen.bmj.com/site/about/guidelines.xhtml

To Russia with anaphylaxis: National survey of physicians knowledge of diagnosis and management of food induced anaphylaxis

Daniel Munblit [1,2,4*], Marina Treneva [3,4], Ilya Korsunskiy [1], Alan Asmanov [3], Alexander Pampura [3,4], John O Warner [2,4]

[1] Faculty of Pediatrics, I. M. Sechenov First Moscow State Medical University, Russia
[2] Department of Paediatrics, Imperial College London, UK
[3] Allergy Department, Veltischev Clinical Pediatric Research Institute of Pirogov Russian National Research Medical University, Russia
[4] International Inflammation (in-FLAME) Network of the World Universities Network

***Corresponding author:**
Dr. Daniel Munblit
Wright Fleming Building
Norfolk Place
London W2 1PG
Tel. +447898257151
Email: daniel.munblit08@imperial.ac.uk

Key words: anaphylaxis, survey, food-induced anaphylaxis, anaphylaxis knowledge

Running Title: Physicians knowledge of diagnosis and management of food induced anaphylaxis in Russia

Funding Source: The authors have no funding relationships relevant to the article to disclose.

Financial Disclosure: The authors have no financial relationships relevant to the article to disclose.

Conflict of Interest: The authors have no conflicts of interest to disclose.

Word count: 2445

Abstract

Objectives: Food allergy is a major burden worldwide and a common problem within paediatric populations, affecting 5-8% of children. Anaphylaxis caused by food proteins is a potentially life-threatening condition and all healthcare practitioners should be aware of its recognition and management. Russia is the largest country in Europe but it is still unknown whether physicians are well prepared to diagnose and manage food induced anaphylaxis effectively. We aimed to examine physicians' knowledge of diagnosis and management of food induced anaphylaxis.

Setting, population and outcomes: A survey was designed and published online at VrachiRF.ru website (for registered Russian-speaking practicing physicians). We obtained information on respondents' clinical settings, experience and specialty. Survey questions were based upon a

For peer review only

characteristic clinical scenario of anaphylaxis due to food ingestion. Outcome measures consisted of correct answers to the anaphylaxis diagnosis and management questions.

Results: Out of total 707 of physicians accessed the survey, 315 (45%) responded to the clinical scenario. 16 respondents reported training in allergy-immunology and have been excluded from the analysis, leaving the final sample size of 299. Respondents were adult physicians (32%) and paediatricians (68%). Overall, 100 (33%) of respondents diagnosed anaphylaxis and only 29% of those making the right diagnosis administered adrenalin (1:1000) IM. Respondents working in secondary/tertiary clinics diagnosed anaphylaxis significantly more often (p=0.04) when compared to primary care/private practice physicians. This difference was also apparent as the the most important influence on responses in the multivariate analysis.

Conclusions: In this national sample of Russian physicians we found poor knowledge in both, anaphylaxis diagnosis and management. Our data shows that the chance of being properly diagnosed with anaphylaxis is 33% and being treated with adrenalin is 10% respectively. These findings highlight lack of anaphylaxis knowledge amongst Russian physicians, both paediatricians and adult specialists and illustrates a need for allergy/anaphylaxis training.

Strenghts and limitations of this study

- Strengths of this survey include the large number of respondents from different medical backgrounds. This is the first survey of anaphylaxis knowledge in Russian physicians and provides an insight into current ability of Russian doctors to diagnose and manage anaphylaxis. As all surveyed physicians answered the same set of questions it allows for comparisons of their knowledge between the groups.
- Limitations of the study include use of non-standardised and validated survey instruments and distribution via VrachiRF.ru website thus respondents may be more educated on various medical topics than average physician.

For peer review only

Introduction

Allergic conditions are a major problem worldwide and their prevalence have increased considerably in the recent decades with a quarter of school-age children in Europe being allergic. Among allergic diseases, food allergy (FA) has become a significant burden with 6-8% of children under the age of three having allergy to at least one food [1] withdetrimental effects on the quality of life [2].

Anaphylaxis is a 'severe, life-threatening generalised or systemic hypersensitivity reaction' [3]. Details on anaphylaxis prevalence, main triggers and mortality rates are available in some countries, but missing in many geographical locations. Recent meta-analysis undertaken by the European Academy of Allergy and Clinical Immunology (EAACI) provides an estimated pooled prevalence of anaphylaxis as 0.3% [4]. The Russian Federation is still a grey area and no data on anaphylaxis prevalence and/or mortality rates are available. The reasons for the lack of research in this field in Russia remain unclear but may be partially explained by lack of a unified digital registry of anaphylaxis events and unavailability of adrenaline autoinjectors, as non of the existing brands are registered in Russian Federation. The registration is a necessary prerequisite for drug to be approved for clinical use. This process includes drug quality, efficacy, safety and, in case with autoinjectors, delivery, assessment by an authorised state body which results in approval for medical use of a drug in the Russian Federation.

Food allergy is a major health concern and is increasing in prevalence reaching an overall pooled point prevalence of 0.9% when confirmed by oral food challenge [5]. Data from the United States shows that food induced anaphylaxis is a cause of 2000 hospitlisations and 200 death cases a year [6]. Food is the leading cause of anaphylactic reactions, especially in paediatric population [7] [8] and is associated with an increased risk of death in patients with co-existing asthma [9]. Coexistentence of food allergy and asthma is not an unusual event as 4-8% of asthmatic children with asthma have food allergy, and more than a third of children with food allergy have asthma [10]. The most recent UK-wide investigation on asthma deaths found that 195 people died of anaphylaxis with co-existant asthma between 2012 and 2013 [11].

EAACI has underlined the importance of anaphylaxis recognition as a clinical emergency, and highlighted the need for healthcare professionals to be aware of it's diagnosis and management [3]. The EAACI Taskforce on anaphylaxis drafted guidelines covering all aspects of recognition,

For peer review only

risk factor assessment, and the management of patients. Despite these efforts, levels of anaphylaxis recognition and management knowledge is still far from perfect [6,12].

The Russian Association of Allergy and Clinical Immnuology (RAACI) local guidelines provide physicians with the basic principles of anaphylaxis diagnosis and management [13] but the degree of anaphylaxis understanding amongst Russian physicians is unknown. Our survey aimed to provide information of Russian physicians knowledge of anaphylaxis diagnosis and management.

Methods

Study design and population

A cross-sectional study was conducted in January 2016 using online survey published on the VrachiRF (vrachirf.ru) website. VrachiRF is a Russian website for registered physicians which provides clinically relevant information and latest news in the medical field. VrachiRF posted a survey between February and March 2016, responses were collected and anonymised data presented in an Excel spreadsheet. The study population included paediatricians and adult physicians.

Survey questionnaire

We developed a case based survey in Russian to evaluate physicians knowledge of anaphylaxis diagnosis and management. The survey consisted of a clinical scenario; open-ended question on diagnosis; multiple choice questions on treatment, required clinical tests and recommendations. The clinical scenario described an anaphylaxic event with multiple systems involvement:

During a busy weekend A&E shift, you are seeing a 6-year-old boy. His main complaint at admission is syncope. 10 minutes after eating a Chinese meal at a friend's house he developed an urticarial rash on the neck. 5 minutes after the rash appearance he complained of abdominal cramping and vomited twice. After 15 minutes, he felt dizzy, developed a wheeze and fainted. He was delivered to the hospital 35 minutes after occurrence of the first symptoms. It is not known if he has food allergy but he had 2 hospitalizations for asthma in the last year.

On examination, his heart rate is 90, blood pressure 90/45mmHg, respiratory rate 22, and oxygen saturation 94%. His weight is 22 kg. He has diffuse wheezing and is in moderate

For peer review only

respiratory distress. He was not carrying his salbutamol inhaler and hasn't received any medications yet.

As no validated survey instrument exists the development of this questionnaire was based on allergy expert opinion and previously published data. The survey was developed by the research team, consisting of paediatricians and allergy specialists. It was pilot tested with external adult physicians, pediatricians and allergy specialists for clarity, prior to its use.

The first question asked responders to provide the most likely diagnosis for the clinical scenario in an open-answer form. The following questions focused on patient management and additional tests needed were presented in the form of a multiple choice. Additional information on participants included age, gender, years of clinical experience, speciality, country and city of residence, clinical practice settings.

Statistical analysis

Data were imported from the survey website into Microsoft Excel 2013, cleaned to detect any missing or invalid variables and then converted into SPSS database. Statistical analysis was performed using IBM SPSS Statistics for Windows, Version 22.0 (Armonk, NY: IBM Corp.).

Statistical significance between the groups have been assessed using x^2 for categorical variables and Mann – Whitney U test for continuous variables. In order to evaluate association between the demographic factors and ability to make the right diagnosis and treatment, logistic regression was used. Results were considered significant when p-values were reported at a level less than 0.05.

For peer review only

Results

Characteristics of the study population

The study was conducted using an online survey questionnaire tool. Out of total 707 of physicians accessed the survey, 315 (45%) responded to the clinical scenario and answered all the questions. 16 respondents reported training in allergy-immunology and have been excluded from the analysis, as we were not aiming to survey specialists, leaving the final sample size of 299.

The study population were predominantly women (85%), in their fifties (mean age of 47.7 years), with two thirds (68%) of respondents working in the paediatric field. More than half (57%) of the surveyed physicians work in primary care settings. 70% of the participants had practiced for more than 15 years. The demographic characteristics of study participants are summarized in Table 1.

Characteristic	N (%)
Gender	
Male	44 (15%)
Female	255 (85%)
Age	47.7 (10.7)ǂ
Specialty	
Paediatrics	203 (68%)
Other	96 (32%)
Clinical settings	
Primary care	169 (57%)
Secondary care	67 (22%)
Tertiary care	36 (12%)
Private practice only	27 (9%)
Clinical experience	
1-5 years	29 (10%)
6-10 years	31 (10%)
11-15 years	30 (10%)
More than 15 years	209 (70%)

Table 1 Characteristics of the survey respondents. ǂ - mean (SD)

For peer review only

Respondents knowledge of anaphylaxis diagnosis and management

Thirty three percent of surveyed physicians diagnosed the presented patient with anaphylaxis. The most popular choice for first line treatment options was IM prednisolone (37%) followed by chloropyramine (26%), with only 15% of respondents agreeing on adrenalin (1:1000) IM as a treatment of choice. Of those making the right diagnosis 29% selected adrenalin as a first-line treatment (Figure 1). Fourty six percent of surveyed physicians considered salbutamol use as appropriate and 47% selected supplemental oxygen as a part of the necessary treatment. Data on respondents knowledge is presented in Table 2.

**Outcomes**	**Responses**	**N=299**<br>**N (%)**
Diagnosis		
What is the most likely diagnosis?	Anaphylaxis	99 (33)
	Not anaphylaxis	200 (67)
Treatment		
Use adrenalin 1:1000 IM as a first line treatment	Yes	45 (15)
	No	254 (85)
Use adrenalin 1:10000 IM as a first line treatment	Yes	31 (10)
	No	268 (90)
Use chloropyramine as a first line treatment	Yes	79 (26)
	No	220 (74)
Use prednisolone IM as a first line treatment	Yes	111 (37)
	No	188 (63)
Uses prednisolone IM in addition to other therapy	Yes	109 (37)
	No	190 (63)
Oxygen supply	Yes	141 (47)
	No	158 (53)
Salbutamol inhaler	Yes	139 (46)
	No	160 (54)
Antihistamine (cetirizine)	Yes	11 (4)
	No	288 (96)
Inhaled steroids	Yes	110 (37)
	No	189 (63)
Further invesigations needed		
Serum tryptase	Yes	69 (23)
	No	230 (77)
Spirometry	Yes	199 (67)
	No	100 (33)
Chest X-ray	Yes	144 (48)
	No	155 (52)
SPT or sIgE testing to food allergens	Yes	213 (71)
	No	86 (29)
sIgG testing to food allergens	Yes	187 (63)
	No	112 (37)
Stool ova and parasite exam	Yes	96 (32)
	No	203 (78)

Table 2 Respondents answers on anaphylaxis Clinical scenario.

For peer review only

48 and 67% selected chest X-ray and spirometry respectively, as required investigations. Most of the respondents considered sIgE (71%) and sIgG (63%) to food allergens testing as necessary tests and 23% suggested serum tryptase measurement

Respondents working in secondary/tertiary clinics diagnosed patient with anaphylaxis significantly more often (p=0.04) when compared to primary care/private practice physicians. This was also the most important factor in the regression model, upon adjustment for participants sex, age, specialty and clinical experience. Neither clinical experience, nor specialty significantly influenced participants decision to use adrenalin. The medication choice was not associated with the participant characteristics (Table 3).

Respondents characteristic	Diagnosed patient with anaphylaxis, %	P value	Used adrenalin 1:1000 IM, %	P value	Used prednisolone IM, %	P value	Used chloropyramine, %	P value
Clinical settings								
Primary care/Private practice	29	**0.04**	13	0.23	41	0.07	27	0.54
Secondary and/or Tertiary care	41		18		30		24	
Clinical experience								
1-10 years	32	0.79	15	0.99	38	0.83	28	0.71
>10 years	34		15		37		26	
Specialty								
Paediatrics	34	0.46	16	0.40	39	0.23	29	0.22
Adult medicine	30		13		32		22	

Table 3 Diagnosis and first line treatment amongst different respondent groups. x^2 test is used for the group comparison. statistically significant difference (p<0.05) appear in bold.

Multivariate analysis outcomes (Table 4) confirmed univariate analysis results showing that clinical setting is the most important factor influencing physicians ability to diagnose anaphylaxis. Those working in primary care/private practice make the correct diagnosis less often than in secondary/tertiary care hospitals OR 0.51 (95% CI 0.30 – 0.86). Participants diagnosing anaphylaxis were much more prone to treat patient with adrenalin OR 5.65 (95% CI 2.81 – 11.36). Prednisolone use was independent of participant characteristics. Two factors influencing chloropyramine prescription were age OR 0.97 (95% CI 0.93 – 0.99) and ability to diagnose anaphylaxis OR 0.35 (95% CI 0.19 – 0.67), respectively.

For peer review only

	Anaphylaxis diagnosis Odds Ratio (95% CI)	P Value	IM Adrenalin Odds Ratio (95% CI)	P Value	Prednisolone Odds Ratio (95% CI)	P Value	Chloropyramine Odds Ratio (95% CI)	P Value
Sex	0.75 (0.36 – 1.57)	0.45	0.71 (0.24 – 2.10)	0.54	1.69 (0.85 – 3.83)	0.14	1.49 (0.70 – 3.15)	0.30
Age	0.97 (0.95 – 1.00)	0.10	0.98 (0.94 – 1.03)	0.42	1.00 (0.98 – 1.04)	0.61	0.97 (0.93 – 0.99)	**0.04**
Specialty	1.34 (0.77 – 2.35)	0.31	1.30 (0.60 – 2.83)	0.51	1.43 (0.82 – 2.50)	0.20	1.59 (0.85 – 2.97)	0.15
Clinical settings	0.51 (0.30 – 0.86)	**0.01**	0.74 (0.36 – 1.50)	0.40	1.55 (0.90 – 2.68)	0.11	0.95 (0.52 – 1.73)	0.86
Clinical experience	1.33 (0.59 – 2.99)	0.49	1.39 (0.45 – 4.31)	0.57	0.94 (0.43 – 2.05)	0.88	1.93 (0.81 – 4.61)	0.14
Anaphylaxis diagnosis	NA	NA	5.65 (2.81 – 11.36)	**<0.01**	0.73 (0.44 – 1.24)	0.24	0.35 (0.19 – 0.67)	**<0.01**

Table 4 Multivariate predictive model for factors influencing physicians decision on anaphylaxis diagnosis and first line management choice. Statistically significant difference (p<0.05) appear in bold.

For peer review only

Discussion

Anaphylaxis awareness has received more attention over the last decade. The findings of our study provide the first insight into knowledge of anaphylaxis diagnosis and management in Russia. Although recent reports from several countries [14] suggest that lack of knowledge in anaphylaxis diagnosis and management is not "as great as previously published", differing patterns are seen in Russia. Sixty seven percent of respondents did not recongise anaphylaxis in a very clearly presented clinical scenario, for which anaphylaxis should be strongly considered. Approximately one in ten professionals made an appropriate choice of adrenalin as a first line treatment and only a third of those who rightfully diagnosed anaphylaxis provided appropriate medication. This suggeststhat food allergic patients could be at increased risk of fatal reaction due to anaphylaxis and this danger is even higher considering non-existence of adrenalin autoinjectors in Russia.

Overall, only ten percent of surveyed physicians were able to both diagnose and provide appropriate treatment to a child with food-induced anaphylaxis, when assessed using a clinical scenario survey instrument. These numbers are much lower than in any of the previous studies up to date [6 14 15].

Two studies done in US included anaphylaxis scenarios with no skin rashes present [6 12] as it is known that anaphylaxis can often maniphest with no obvious cutaneous reaction. In our survey we did not overcomplicate the clinical scenario, providing respondents with a combination of the cutaneous, cardiorespiratory, GI and generalized symptoms. While the majority of the physicians recognised allergic reaction in the clinical scenario, only 33% of respondents diagnosed patient with anaphylaxis. This outcome shows that most of the doctors are not be aware of the anaphylaxis diagnostic criteria. Our clinical scenario clearly meets anaphylaxis diagnostic criteria stated within EAACI [3] and RAACI [13] guidelines. Poor EAACI guidelines awareness in Russia may be a result of generally low level of English but unawareness of RAACI guidelines is related to poor distribution and promotion of this document.

Anaphylaxis recognition is the most important prerequisite for successful management, as physicians are more likely to administer adrenalin if they have diagnosed patient with anaphylaxis, as reported previously [6]. Our data is an agreement with the outcomes of the previous studies. Among the factors potentially impacting participants knowledge we did not

For peer review only - http://bmjopen.bmj.com/site/about/guidelines.xhtml

find any significant influence of primary specialty and/or clinical experience on physicians ability to diagnose anaphylaxis. Respondents working in the secondary or tertiary centers possess better knowledge on anaphylaxis diagnosis which maybe can be explained by their daily exposure to highly specialised medical environment and involvement in academic activities.

When potential factors influencing respondent decision making has been reviewed in multivariate analysis we confirmed that clinical settingshad the biggest impact on anaphylaxis diagnosis but not first line treatment. Adrenalin use is strongly dependant on initial diagnosis and if physician diagnosed the patient with anaphylaxis, it was much more likely that an appropriate medicationwas chosen. Chloropyramine use as a first line treatment was inversely associated with anaphylaxis diagnosis.

Intramuscular adrenalin is the drug of choice in the scenario of an anaphylactic event and should be injected as soon as possible. It is evident that most of the respondents preferred intramuscular prednisolone or oral chloropyramine use, irrespective of their speciality, clinical experience and/or clinical settings. Prednisolone or other corticosteroids can be used for biphasic anaphylaxis prevention, which accounts for around 20% of anaphylactic cases [16], and may reduce the risk of late-phase respiratory symptoms [3], but it does not resolve acute anaphylaxis. First generation antihistamines are not an optimal choice for anaphylaxis management, especially when patient is lethargic, as they may cause drowsiness masking respiratory symptoms [13].

Beta-2 agonists can be used during anaphylaxis to assist in the event of bronchoconstriction and the patient should be provided with an oxygen supply [3] but only half of respondents selected these options as a part of management required.

Surveyed physicians selected some further investigations following the anaphylactic event. The majority of respondents choose SPT/sIgE and/or sIgG testing to food allergens, to detect a possible trigger of reaction. The number of physicians prioritising SPT/sIgE over sIgG testing to food was little different. These results suggest lack of differentiation between sIgE and sIgG amongst Russian physicians. Recently, a number of position papers has been released, warning practicioners against IgG testing use in the diagnosis of food allergy [17-19] but these papers are not easily available in Russia and no relevant statements has been provided by RAACI. Approximately one in four respondents considered serum tryptase as a useful test in the

For peer review only

described event. Routine evaluation of serum tryptase levels is still a grey area but recent data suggests that it is particularly useful in severe anaphylactic events due to drugs and wasp.bee venom reactions. However, tryptase is not be raised in many cases of food induced other than if milk is a causative allergen [20].

There were a number of limitations to the study. The survey is not standardised and validated. It was distributed via VrachiRF website which can be considered as one of the most popular sources for Russian physicians. Usually, people using medical websites possess better knowledge on a wide range of medical topics, meaning that survey outcomes do not necessarily provide an image of an average doctor practicing in this country. Lack of correct answers does not always automatically translate to wrong clinical decisions in a real life scenario. As survey was presented in Russian, results can not be generalised to medical professionals living in other countries. However, we believe that our results are closer to a best case interpretation and therefore are all the more concerning.

Although limitations are clear, results of this survey suggest a prominent deficit of knowledge on anaphylaxis diagnosis and management in Russia. Despite anaphylaxis guidelines being easily accessible via EAACI and RAACI websites they are not promoted well enough among Russian health professionals which results in poor knowledge. Our data highlights the need for educational programme development and changes to the undergraduate medical education curriculum. This should lead to a better care quality for children with food allergy at risk of anaphylactic events and may potentially reduce morbidity and mortality. Furthermore, detection of existing gaps in anaphylaxis diagnosis and management is highly important [14] at a time when food allergy is on the rise, particularly in the paediatric population.

Acknowledgments

We would like to thank physicians that kindly agreed to complete the survey and Vrachirf.ru website for their help with the survey.

Contributorship statement

DM, MT, IK and JW designed a questionnaire. AA and AP provided an independent evaluation of the survey instrument and assisted with the pre-survey pilot testing. DM and MT conceived the study, designed the methods and coordinated data collection. DM wrote the first draft of the

For peer review only

paper. MT, JW contributed to the writing of the paper. All authors critically revised the manuscript and approved the final version.

Competing interests

None declared.

Funding

This research received no specific grant from any funding agency in the public, commercial, or not-for-profit sectors.

Author Data sharing statement

The Excel data set is available from corresponding author on request.

References

1. National Institute for Health and Care Excellence (NICE). Food allergy. London: NICE, 2016.
2. Flokstra-de Blok BM, Dubois AE, Vlieg-Boerstra BJ, et al. Health-related quality of life of food allergic patients: comparison with the general population and other diseases. *Allergy* 2010;65(2):238-44. doi: 10.1111/j.1398-9995.2009.02121.x
3. Muraro A, Roberts G, Worm M, et al. Anaphylaxis: guidelines from the European Academy of Allergy and Clinical Immunology. *Allergy* 2014;69(8):1026-45. doi: 10.1111/all.12437
4. Panesar SS, Javad S, de Silva D, et al. The epidemiology of anaphylaxis in Europe: a systematic review. *Allergy* 2013;68(11):1353-61. doi: 10.1111/all.12272
5. Nwaru BI, Hickstein L, Panesar SS, et al. The epidemiology of food allergy in Europe: a systematic review and meta-analysis. *Allergy* 2014;69(1):62-75. doi: 10.1111/all.12305
6. Krugman SD, Chiaramonte DR, Matsui EC. Diagnosis and management of food-induced anaphylaxis: a national survey of pediatricians. *Pediatrics* 2006;118(3):e554-60. doi: 10.1542/peds.2005-2906
7. de Silva IL, Mehr SS, Tey D, et al. Paediatric anaphylaxis: a 5 year retrospective review. *Allergy* 2008;63(8):1071-6. doi: 10.1111/j.1398-9995.2008.01719.x
8. Hompes S, Kohli A, Nemat K, et al. Provoking allergens and treatment of anaphylaxis in children and adolescents--data from the anaphylaxis registry of German-speaking countries. *Pediatr Allergy Immunol* 2011;22(6):568-74. doi: 10.1111/j.1399-3038.2011.01154.x
9. Vetander M, Helander D, Flodstrom C, et al. Anaphylaxis and reactions to foods in children--a population-based case study of emergency department visits. *Clin Exp Allergy* 2012;42(4):568-77. doi: 10.1111/j.1365-2222.2011.03954.x
10. Simpson AB, Glutting J, Yousef E. Food allergy and asthma morbidity in children. *Pediatr Pulmonol* 2007;42(6):489-95. doi: 10.1002/ppul.20605
11. Physicians RCo. Why asthma still kills: the National Review of Asthma Deaths (NRAD) Confidential Enquiry report. . London: Royal College of Physicians, 2014.
12. Altman AM, Camargo CA, Jr., Simons FE, et al. Anaphylaxis in America: A national physician survey. *J Allergy Clin Immunol* 2015;135(3):830-3. doi: 10.1016/j.jaci.2014.10.049
13. Russian Association of Allergy and Clinical Immunology (RACI). Federal Clinical Recommendations on Anaphylactic Shock. 2013:1-13.

14. Wang J, Young MC, Nowak-Wegrzyn A. International survey of knowledge of food-induced anaphylaxis. *Pediatr Allergy Immunol* 2014;25(7):644-50. doi: 10.1111/pai.12284
15. Wang J, Sicherer SH, Nowak-Wegrzyn A. Primary care physicians' approach to food-induced anaphylaxis: a survey. *J Allergy Clin Immunol* 2004;114(3):689-91.
16. Tole JW, Lieberman P. Biphasic anaphylaxis: review of incidence, clinical predictors, and observation recommendations. *Immunol Allergy Clin North Am* 2007;27(2):309-26, viii. doi: 10.1016/j.iac.2007.03.011
17. Stapel SO, Asero R, Ballmer-Weber BK, et al. Testing for IgG4 against foods is not recommended as a diagnostic tool: EAACI Task Force Report. *Allergy* 2008;63(7):793-6. doi: 10.1111/j.1398-9995.2008.01705.x
18. Bock SA. AAAAI support of the EAACI Position Paper on IgG4. *J Allergy Clin Immunol* 2010;125(6):1410. doi: 10.1016/j.jaci.2010.03.013
19. Carr S, Chan E, Lavine E, et al. CSACI Position statement on the testing of food-specific IgG. *Allergy Asthma Clin Immunol* 2012;8(1):12. doi: 10.1186/1710-1492-8-12
20. De Schryver S, Halbrich M, Clarke A, et al. Tryptase levels in children presenting with anaphylaxis: Temporal trends and associated factors. *J Allergy Clin Immunol* 2016;137(4):1138-42. doi: 10.1016/j.jaci.2015.09.001

For peer review only

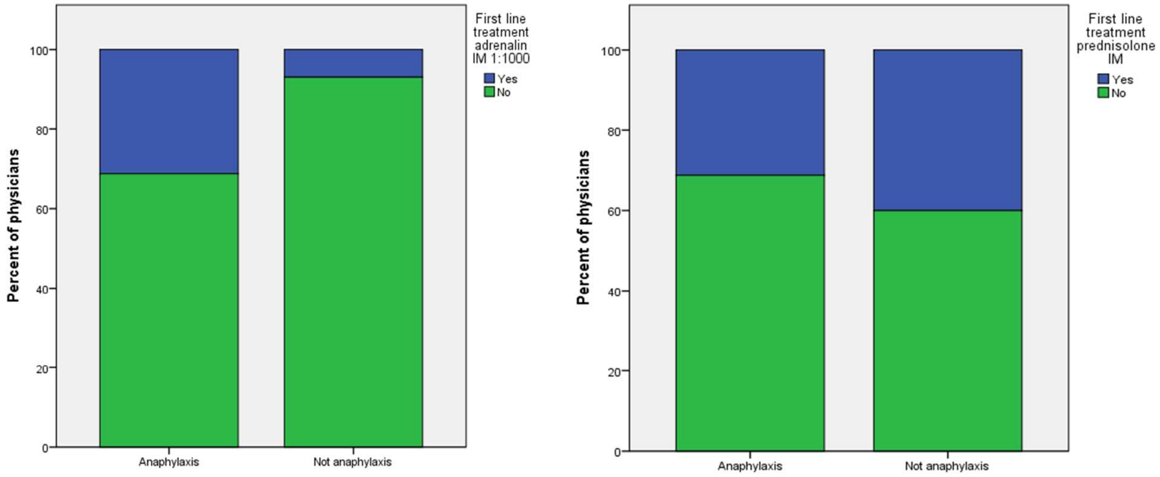

Figure 1 Physicians choice of adrenalin as a first line treatment of anaphylaxis. This graph shows proportion of physicians to choose: IM adrenaline 1:1000 or IM as a first line treatment, depending on their choice of diagnosis.

For peer review only

Checklist from the https://www.equator-network.org/reporting-guidelines/good-practice-in-the-conduct-and-reporting-of-survey-research/ as recommended.

When reporting survey research, it is essential that a number of key points are covered (though the length and depth of reporting will be dependent upon journal style). These key points are presented as a ‘checklist’ below:

1. Explain the purpose or aim of the research, with the explicit identification of the research question.	✓
2. Explain why the research was necessary and place the study in context, drawing upon previous work in relevant fields (the literature review).	✓
3. Describe in (proportionate) detail how the research was done.	✓
a. State the chosen research method or methods, and justify why this method was chosen.	✓
b. Describe the research tool. If an existing tool is used, briefly state its psychometric properties and provide references to the original development work. If a new tool is used, you should include an entire section describing the steps undertaken to develop and test the tool, including results of psychometric testing.	✓
c. Describe how the sample was selected and how data were collected, including:	✓
iv.How were potential subjects identified?	✓
v.How many and what type of attempts were made to contact subjects?	✓
vi.Who approached potential subjects?	✓
vii.Where were potential subjects approached?	✓
viii.How was informed consent obtained?	✓
ix.How many agreed to participate?	✓
x.How did those who agreed differ from those who did not agree?<br><br>It is very hard to meet this recommendation due to a study design. Surveyed physicians voluntarily accessed the survey online and data available provides information and details only on those respondents who accessed the survey. We do not know how they differ from those physicians deciding not to undertake the survey.	✗
xi.What was the response rate?	✓
4. Describe and justify the methods and tests used for data analysis.	✓
5. Present the results of the research. The results section should be clear, factual, and concise.	✓
6. Interpret and discuss the findings. This ‘discussion’ section should not simply reiterate results; it should provide the author’s critical reflection upon both the results and the processes of data collection. The discussion should assess how well the study met the research question, should describe the	✓

For peer review only - http://bmjopen.bmj.com/site/about/guidelines.xhtml

problems encountered in the research, and should honestly judge the limitations of the work.	
7. Present conclusions and recommendations.	✓

For peer review only

For peer review only - http://bmjopen.bmj.com/site/about/guidelines.xhtml

BMJ Open

A national survey of Russian physicians' knowledge of diagnosis and management of food induced anaphylaxis

Journal:	*BMJ Open*
Manuscript ID	bmjopen-2017-015901.R1
Article Type:	Research
Date Submitted by the Author:	28-Mar-2017
Complete List of Authors:	Munblit, Daniel; Pervyj Moskovskij gosudarstvennyj medicinskij universitet imeni I M Secenova, Faculty of Pediatrics; Imperial College London, Paediatrics<br>Treneva, Marina; Veltischev Clinical Pediatric Research Institute of Pirogov Russian National Research Medical University; International Inflammation (in-FLAME) Network of the World Universities Network<br>Korsunskiy, Ilya; Pervyj Moskovskij gosudarstvennyj medicinskij universitet imeni I M Secenova, Faculty of Pediatrics<br>Asmanov, Alan; Veltischev Clinical Pediatric Research Institute of Pirogov Russian National Research Medical University<br>Pampura, Alexander; Veltischev Clinical Pediatric Research Institute of Pirogov Russian National Research Medical University; International Inflammation (in-FLAME) Network of the World Universities Network<br>Warner, John O.; Imperial College London, Paediatrics; International Inflammation (in-FLAME) Network of the World Universities Network
**Primary Subject Heading**:	Immunology (including allergy)
Secondary Subject Heading:	Paediatrics
Keywords:	anaphylaxis, survey, food-induced anaphylaxis, anaphylaxis knowledge

SCHOLARONE™
Manuscripts

A national survey of Russian physicians' knowledge of diagnosis and management of food induced anaphylaxis

Daniel Munblit [1,2,4*], Marina Treneva [3,4], Ilya Korsunskiy [2], Alan Asmanov [3], Alexander Pampura [3,4], John O Warner [1,4,5]

[1] Department of Paediatrics, Imperial College London, UK
[2] Faculty of Pediatrics, I. M. Sechenov First Moscow State Medical University, Russia
[3] Allergy Department, Veltischev Clinical Pediatric Research Institute of Pirogov Russian National Research Medical University, Russia
[4] International Inflammation (in-FLAME) Network of the World Universities Network
[5] National Institute of Health Research Collaboration for Leadership in Applied Health Research and Care for NW London, UK

***Corresponding author:**
Dr. Daniel Munblit
Wright Fleming Building
Norfolk Place
London W2 1PG
Tel. +447898257151
Email: daniel.munblit08@imperial.ac.uk

Key words: anaphylaxis, survey, food-induced anaphylaxis, anaphylaxis knowledge

Running Title: Physicians knowledge of diagnosis and management of food induced anaphylaxis in Russia

Funding Source: The authors have no funding relationships relevant to the article to disclose.

Financial Disclosure: The authors have no financial relationships relevant to the article to disclose.

Conflict of Interest: The authors have no conflicts of interest to disclose.

Word count: 2722

Abstract

Objectives: Food allergy is an increasing burden worldwide and is a common problem within paediatric populations, affecting 5-8% of children. Anaphylaxis caused by food proteins is a potentially life-threatening condition and all healthcare practitioners should be aware of its recognition and management. Russia is the largest country in Europe but it is still unknown whether physicians are prepared to diagnose and manage food induced anaphylaxis effectively. We aimed to examine physicians' knowledge of diagnosis and management of food induced anaphylaxis.

For peer review only

Setting, population and outcomes: A survey was designed and published online at VrachiRF.ru website (for registered Russian-speaking practicing physicians). We obtained information on respondents' clinical settings, experience and specialty. Survey questions were based upon a characteristic clinical scenario of anaphylaxis due to food ingestion. Outcome measures consisted of correct answers to the anaphylaxis diagnosis and management questions.

Results: From a total of 707 of physicians accessed in the survey, 315 (45%) responded to the clinical scenario. 16 respondents reported training in allergy-immunology and have been excluded from the analysis, leaving the final sample size of 299. Respondents were paediatricians (68%) and other specialities adult physicians (32%). Overall, 100 (33%) of respondents diagnosed anaphylaxis but only 29% of those making the correctdiagnosis administered adrenalin (1:1000) IM. Respondents working in secondary/tertiary clinics diagnosed anaphylaxis significantly more often (p=0.04) when compared to primary care/private practice physicians. This difference was also apparent as the most important influence on responses in the multivariate analysis.

Conclusions: In this national sample of Russian physicians we found poor knowledge in both, anaphylaxis diagnosis and management. Our data shows that the chance of being properly diagnosed with anaphylaxis is 33% and being appropriately treated with adrenalin is 10% respectively. These findings highlight lack of anaphylaxis knowledge amongst Russian physicians, both paediatricians and other specialists and illustrates the urgent need for allergy/anaphylaxis training.

Strengths and limitations of this study

- Strengths of this survey include the large number of respondents from different medical backgrounds. This is the first survey of anaphylaxis knowledge in Russian physicians and provides an insight into current ability of Russian doctors to diagnose and manage anaphylaxis. As all surveyed physicians answered the same set of questions it allows for comparisons of their knowledge between the groups.
- Limitations of the study are the use of non-standardised and validated survey instruments.The distribution via VrachiRF.ru website where respondents may be more educated than average physicians, and the 45% response rate probably present a best rather than worst case scenario.

For peer review only

Introduction

Allergic conditions are a major problem worldwide and their prevalence have increased considerably in the recent decades with a quarter of school-age children in Europe being allergic. Among allergic diseases, food allergy (FA) has become a significant burden with 6-8% of children under the age of three having allergy to at least one food [1]. FA has detrimental effects on the quality of life [2] and health/economics with prescribing of adrenalin auto-injectors (AAIs) having risen over 350 fold in the UK from 2000 to 2012 [3].

Anaphylaxis is a 'severe, life-threatening generalised systemic hypersensitivity reaction' [4]. Details on anaphylaxis prevalence, the main triggers and mortality rates are available in some countries, but missing in many geographical locations. A recent meta-analysis undertaken by the European Academy of Allergy and Clinical Immunology (EAACI) provided an estimated pooled prevalence of anaphylaxis of 0.3% [5]. No data on anaphylaxis prevalence and/or mortality rates are available from the Russian Federation. The reasons for the lack of research in this field in Russia remain unclear but may be partially explained by lack of a unified digital registry of anaphylaxis events and unavailability of AAIs, as none of the existing brands are registered in Russian Federation. The registration is a necessary prerequisite for a drug to be approved for clinical use. The process of approval for medical use of a drug includes drug quality, efficacy, safety and, in case with autoinjectors, delivery, assessment by an authorised state body.

Food allergy is a major health concern and is increasing in prevalence reaching an overall pooled point prevalence of 0.9% when confirmed by oral food challenge [6]. Data from the United States shows that food induced anaphylaxis is a cause of 2000 hospitalisations and 200 death a year [7]. Food is the leading cause of anaphylactic reactions, especially in paediatric populations [8 9] and when associated with co-existing asthma has an increased risk of death[10]. Coexistence of food allergy and asthma is not an unusual event as 4-8% of asthmatic children with asthma have food allergy, and more than a third of children with food allergy have asthma [11]. The most recent UK-wide investigation on 195 asthma deaths found that many people died of asthma with co-existant allergy between 2012 and 2013 [12].

EAACI has underlined the importance of anaphylaxis recognition as a clinical emergency, and highlighted the need for healthcare professionals to be aware of it's diagnosis and management

For peer review only

[4]. The EAACI Taskforce on anaphylaxis drafted guidelines covering all aspects of recognition, risk factor assessment, and the management of patients. Despite these efforts, levels of anaphylaxis recognition and management knowledge is still far from perfect [7 13].

The Russian Association of Allergy and Clinical Immunology (RAACI) local guidelines provide physicians with the basic principles of anaphylaxis diagnosis and management [14 15] but the degree of anaphylaxis understanding amongst Russian physicians is unknown. Our survey aimed to provide information of Russian physicians knowledge of anaphylaxis diagnosis and management.

Methods

Study design and population

A cross-sectional study was conducted in January 2016 using an online survey published on the VrachiRF (vrachirf.ru) website. VrachiRF is a popular Russian professional website for registered physicians (with about half a million subscribers) which provides clinically relevant information and latest news in the medical field. The website sends daily emails to all the members of VrachiRF community with the latest news and updates and a special email on Sunday, providing members with the top news of the week. VrachiRF posted a survey for 4 weeks (between February and March 2016), responses were collected and anonymised data presented in an Excel spreadsheet. The study population included paediatricians and adult physicians of various specialities. The survey was conducted following ethical approval by Moscow Clinical Hospital №9, named after G.N.Speransky, Ethics committee.

Survey questionnaire

We developed a case based survey in Russian to evaluate physicians knowledge of anaphylaxis diagnosis and management. The survey consisted of a clinical scenario; open-ended questions on diagnosis; multiple choice questions on treatment, required clinical tests and recommendations. The clinical scenario described an anaphylaxic event with multiple system involvement:

During a busy weekend A&E shift, you are seeing a 6-year-old boy. His main complaint at admission is syncope. 10 minutes after eating a Chinese meal at a friend's house he developed an urticarial rash on the neck. 5 minutes after the rash appearance he complained of abdominal

For peer review only

cramping and vomited twice. After 15 minutes, he felt dizzy, developed a wheeze and fainted. He was delivered to the hospital 35 minutes after occurrence of the first symptoms. It is not known if he has food allergy but he had 2 hospitalizations for asthma in the last year.

On examination, his heart rate is 90, blood pressure 90/45mmHg, respiratory rate 22, and oxygen saturation 94%. His weight is 22 kg. He has diffuse wheezing and is in moderate respiratory distress. He was not carrying his salbutamol inhaler and hasn't received any medications yet.

As no validated survey instrument exists the development of this questionnaire was based on allergy expert opinion and previously published data. The survey was developed by the research team, consisting of paediatricians and allergy specialists. It was pilot tested with external adult physicians, paediatricians and allergy specialists for clarity, prior to its use.

The first question asked responders to provide the most likely diagnosis for the clinical scenario in an open-answer form. The next page of online survey provided respondents with additional information on the patient: *"on further questioning when the boy had recovered, he reported that the Chinese meal contained egg fried rice, beef, bean shoots, sesame, cashew nuts and peanuts. He has previously sometimes complained of itchy throat after eating chocolate and he had eczema in infancy worsened when eating egg. However, he has recently had egg without any reaction."*The following questions focused on patient management and additional investigations required which were presented in the form of a multiple choice. Additional information on participants included age, gender, years of clinical experience, speciality, country and city of residence, clinical practice settings. Full version of the survey is available as a supplementary document 1.

Statistical analysis

Data were imported from the survey website into Microsoft Excel 2013, cleaned to detect any missing or invalid variables and then converted into the SPSS database. Statistical analysis was performed using IBM SPSS Statistics for Windows, Version 22.0 (Armonk, NY: IBM Corp.).

Statistical significance between the groups have been assessed using x^2 for categorical variables and Mann – Whitney U test for continuous variables. In order to evaluate any association between the demographic factors and ability to make the right diagnosis and

For peer review only

treatment, logistic regression was used. Results were considered significant when p-values were reported at a level less than 0.05.

Results

Characteristics of the study population

The study was conducted using an online survey questionnaire tool. Of the 707 physicians accessed the survey, 315 (45%) responded to the clinical scenario and answered all the questions. 16 respondents reported that they had received training in allergy-immunology and have been excluded from the analysis, as we were not aiming to survey specialists, leaving the final sample size of 299.

The study population were predominantly women (85%), in their fifties (mean age of 47.7 years), with two thirds (68%) of respondents worked in the paediatric field. More than half (57%) of the surveyed physicians work in primary care settings. 70% of the participants had practiced for more than 15 years. The demographic characteristics of study participants and physicians decided not to answer the survey are summarized in Table 1.

Characteristic	N (%) Respondents	N (%) Non-respondents	P value (χ2)
**Gender**			
**Male**	44 (15%)	65 (17%)	
**Female**	255 (85%)	325 (83%)	0.48
**Age**	47.7 (10.7)ɫ	49.0 (9.44)ɫ	0.06
**Specialty**			
**Paediatrics**	203 (68%)	205 (53%)	
**Other**	96 (32%)	185 (47%)	**<0.01**
**Clinical settings**			
**Primary care**	169 (57%)	224 (57%)	
**Secondary care**	67 (22%)	94 (24%)	
**Tertiary care**	36 (12%)	42 (11%)	
**Private practice only**	27 (9%)	32 (8%)	0.92
**Clinical experience**			
**1-5 years**	29 (10%)	25 (6%)	
**6-10 years**	31 (10%)	33 (9%)	
**11-15 years**	30 (10%)	54 (14%)	
**More than 15 years**	209 (70%)	278 (71%)	0.17

Table 1 Characteristics of the survey respondents and non-respondents. ɫ - mean (SD)

For peer review only

Respondents knowledge of anaphylaxis diagnosis and management

Thirty three percent of surveyed physicians diagnosed the presented patient with anaphylaxis. The most popular choice for first line treatment options was IM prednisolone (37%) followed by chloropyramine (26%), with only 15% of respondents suggested adrenalin (1:1000) IM as a treatment of choice. Of those making the correct diagnosis only 29% selected adrenalin as a first-line treatment (Figure 1). Forty six percent of surveyed physicians considered salbutamol use as appropriate and 47% selected supplemental oxygen as a part of the necessary treatment. Data on respondents knowledge is presented in Table 2.

**Outcomes**	**Responses**	**N=299**<br>**N (%)**
Diagnosis		
**What is the most likely diagnosis?**	Anaphylaxis	99 (33)
	Not anaphylaxis	200 (67)
Treatment		
**Use adrenalin 1:1000 IM as a first line treatment**	Yes	45 (15)
	No	254 (85)
**Use adrenalin 1:10000 IM as a first line treatment**	Yes	31 (10)
	No	268 (90)
**Use chloropyramine as a first line treatment**	Yes	79 (26)
	No	220 (74)
**Use prednisolone IM as a first line treatment**	Yes	111 (37)
	No	188 (63)
**Uses prednisolone IM in addition to other therapy**	Yes	109 (37)
	No	190 (63)
**Oxygen supply**	Yes	141 (47)
	No	158 (53)
**Salbutamol inhaler**	Yes	139 (46)
	No	160 (54)
**Antihistamine (cetirizine)**	Yes	11 (4)
	No	288 (96)
**Inhaled steroids**	Yes	110 (37)
	No	189 (63)
Further invesigations needed		
**Serum tryptase**	Yes	69 (23)
	No	230 (77)
**Spirometry**	Yes	199 (67)
	No	100 (33)
**Chest X-ray**	Yes	144 (48)
	No	155 (52)
**SPT or sIgE testing to food allergens**	Yes	213 (71)
	No	86 (29)
**sIgG testing to food allergens**	Yes	187 (63)
	No	112 (37)
**Stool ova and parasite exam**	Yes	96 (32)
	No	203 (78)

Table 2 Respondents answers on anaphylaxis Clinical scenario.

For peer review only

48 and 67% selected chest X-ray and spirometry respectively, as required investigations. Most of the respondents considered sIgE (71%) and sIgG (63%) to food allergens testing as necessary tests but only 23% suggested serum tryptase measurement

Respondents working in secondary/tertiary clinics diagnosed patient with anaphylaxis significantly more often (p=0.04) when compared to primary care/private practice physicians. This was also the most important factor in the regression model, upon adjustment for participants sex, age, specialty and clinical experience. Neither clinical experience, nor specialty significantly influenced participants decision to use adrenalin. The medication choice was not associated with the participant characteristics (Table 3).

Multivariate analysis outcomes (Table 4) confirmed univariate analysis results showing that clinical setting is the most important factor influencing physicians ability to diagnose anaphylaxis. Those working in primary care/private practice make the correct diagnosis less often than in secondary/tertiary care hospitals OR 0.51 (95% CI 0.30 – 0.86). Participants diagnosing anaphylaxis were much more likely to treat patient with adrenalin OR 5.65 (95% CI 2.81 – 11.36). Prednisolone use was independent of participant characteristics. Two factors influencing chloropyramine prescription were age (younger age decreased the likelihood of prescribing) OR 0.97 (95% CI 0.93 – 0.99) and ability to diagnose anaphylaxis (right diagnosis decreased the likelihood of prescribing) OR 0.35 (95% CI 0.19 – 0.67), respectively.

For peer review only

Respondents characteristic	Diagnosed patient with anaphylaxis, %	OR (95% CI)	P value	Used adrenalin 1:1000 IM, %	OR (95% CI)	P value	Used prednisolone IM, %	OR (95% CI)	P value	Used chloropyramine, %	OR (95% CI)	P value
Clinical settings												
Primary care/Private practice	29	0.59 (0.36 - 0.98)	**0.04**	13	0.68 (0.35-1.29)	0.23	41	1.60 (0.96-2.66)	0.07	27	1.18 (0.69-2.05	0.54
Secondary and/or Tertiary care	41			18			30			24		
Clinical experience												
1-10 years	32	0.92 (0.50-1.69)	0.79	15	0.99 (0.45-2.20)	0.99	38	1.07 (0.60-1.91)	0.83	28	1.13 (0.60-2.12)	0.71
>10 years	34			15			37			26		
Specialty												
Paediatrics	34	1.22 (0.72-2.05)	0.46	16	1.36 (0.67-2.77)	0.40	39	1.36 (0.82-2.28)	0.23	29	1.43 (0.81-2.53)	0.22
Other specialities	30			13			32			22		

Table 3 Diagnosis and first line treatment amongst different respondent groups. x^2 test is used for the group comparison. Statistically significant difference (p<0.05) appear in bold.

	Anaphylaxis diagnosis Odds Ratio (95% CI)	P Value	IM Adrenalin Odds Ratio (95% CI)	P Value	Prednisolone Odds Ratio (95% CI)	P Value	Chloropyramine Odds Ratio (95% CI)	P Value
Sex	0.75 (0.36 – 1.57)	0.45	0.71 (0.24 – 2.10)	0.54	1.69 (0.85 – 3.83)	0.14	1.49 (0.70 – 3.15)	0.30
Age	0.97 (0.95 – 1.00)	0.10	0.98 (0.94 – 1.03)	0.42	1.00 (0.98 – 1.04)	0.61	0.97 (0.93 – 0.99)	**0.04**
Specialty	1.34 (0.77 – 2.35)	0.31	1.30 (0.60 – 2.83)	0.51	1.43 (0.82 – 2.50)	0.20	1.59 (0.85 – 2.97)	0.15
Clinical settings	0.51 (0.30 – 0.86)	**0.01**	0.74 (0.36 – 1.50)	0.40	1.55 (0.90 – 2.68)	0.11	0.95 (0.52 – 1.73)	0.86
Clinical experience	1.33 (0.59 – 2.99)	0.49	1.39 (0.45 – 4.31)	0.57	0.94 (0.43 – 2.05)	0.88	1.93 (0.81 – 4.61)	0.14
Anaphylaxis diagnosis	NA	NA	5.65 (2.81 – 11.36)	**<0.01**	0.73 (0.44 – 1.24)	0.24	0.35 (0.19 – 0.67)	**<0.01**

Table 4 Multivariate predictive model for factors influencing physicians decision on anaphylaxis diagnosis and first line management choice. Statistically significant difference (p<0.05) appear in bold.

For peer review only

Discussion

Anaphylaxis awareness has received more attention over the last decade. The findings of our study provide the first insight into knowledge of anaphylaxis diagnosis and management in Russia. Although recent reports from several countries [16] suggest that lack of knowledge in anaphylaxis diagnosis and management is not "as great as previously published", differing patterns are seen in Russia. Sixty seven percent of respondents did not recognise anaphylaxis in a very clearly presented clinical scenario, for which anaphylaxis should be strongly considered. Approximately one in ten professionals made an appropriate choice of adrenalin as a first line treatment and only a third of those who rightfully diagnosed anaphylaxis provided appropriate medication. Given a response rate of 45%, our results are more likely to represent a best rather than worst case scenario. It suggests that food allergic patients could be at increased risk of fatal reaction due to anaphylaxis in Russia and this danger is even higher considering lack of availability of AAIsin Russia.

Overall, only ten percent of surveyed physicians were able to both diagnose and provide appropriate treatment for a child with food-induced anaphylaxis, when assessed using a clinical scenario survey instrument. These numbers are much lower than in any of the previous studies up to date [7 16 17], but corresponds well with real-life data, showing that anaphylaxis management is still far from satisfactory based on established recommendations. Analysis of a large anaphylaxis registry showed discrepancies between existing management guidelines and their implementation in clinical practice [18]. Similar outcomes has been recently confirmed from the European Anaphylaxis Registry data-base [19].

Two studies from US included anaphylaxis scenarios with no skin rashes present [7 13] as it is known that anaphylaxis can often manifest with no obvious cutaneous reaction. In our survey, we did not overcomplicate the clinical scenario, providing respondents with a combination of the cutaneous, cardiorespiratory, GI and generalized symptoms. While the majority of the physicians recognised an allergic reaction in the clinical scenario, only 33% of respondents diagnosed the patient as having anaphylaxis. This outcome shows that most of the doctors are not be aware of the anaphylaxis diagnostic criteria. Our clinical scenario clearly meets anaphylaxis diagnostic criteria stated within EAACI [4] and RAACI [14 15] guidelines. Poor EAACI guidelines awareness in Russia may be a result of a generally low level of English

For peer review only

comprehansion but unawareness of RAACI guidelines is likely related to poor distribution and promotion.

Anaphylaxis recognition is the most important prerequisite for successful management, as physicians are more likely to administer adrenalin if they have correctly diagnosed the patient, as reported previously [7]. Our data is an agreement with the outcomes of previous studies. Among the factors potentially impacting participants knowledge we did not find any significant influence of primary specialty and/or clinical experience on physicians ability to diagnose anaphylaxis. Respondents working in the secondary or tertiary centres possess better knowledge on anaphylaxis diagnosis which maybeexplained by their daily exposure to highly specialised medical environment and involvement in academic activities.

Conversely chloropyramine use as a first line treatment was inversely associated with anaphylaxis diagnosis. To what extent this is due to resort to medications which are freely available rather than a lack of knowledge cannot be determined but the latter is more likely given the poor responses to further investigation questions.

Intramuscular adrenalin is the drug of choice in the scenario of an anaphylactic event and should be injected as soon as possible. [18] [19]It is evident that in our survey, most of the respondents preferred intramuscular prednisolone or oral chloropyramine use, irrespective of their speciality, clinical experience and/or clinical settings. Prednisolone or other corticosteroids can be used for biphasic anaphylaxis prevention, which accounts for around 20% of anaphylactic cases [20], and may reduce the risk of late-phase respiratory symptoms [4], but it does not resolve acute anaphylaxis. First generation antihistamines are not an optimal choice for anaphylaxis management, especially when patient is lethargic, as they may cause drowsiness and mask respiratory symptoms [14].

Beta-2 agonists can be used during anaphylaxis to assist in the event of bronchoconstriction and the patients should be provided with supplemental oxygen [4] but only half of respondents selected these options as a part of required management.

The majority of respondents choose SPT/sIgE and/or sIgG testing to food allergens, to detect a possible trigger of reaction. The number of physicians prioritising SPT/sIgE over sIgG testing to food was little different. These results suggest lack of differentiation between sIgE and sIgG

For peer review only

amongst Russian physicians. Recently, a number of position papers has been released, warning practitioners against IgG testing use in the diagnosis of food allergy [21-23] but these papers are not easily available in Russia and no relevant statements has been provided by RAACI. Approximately one in four respondents considered serum tryptase as a useful test in thecase scenario. Routine evaluation of serum tryptase levels is still a grey area but recent data suggests that it is particularly useful in severe anaphylactic events due to drugs and wasp/bee venom reactions. However, tryptase is not be raised in many cases of food induced anaphylaxis other than if milk is a causative allergen [24].

There were several limitations to the study. The survey is not standardised and validated. It was distributed via VrachiRF website which can be considered as one of the most popular sources for Russian physicians. Low response rate is a recognisedissue in survey research, which potentially may lead to selection bias. The response rate in this study was 45% which is not ideal but slightly above average for online surveys [25]. Respondents tended to work in paediatric field more often than non-respondents, probably due to the case scenario describing anaphylaxis in a child.

Clinicians using medical websites and responding to a competence questionaire may possess better knowledge on a wide range of medical topics, meaning that survey outcomes do not necessarily provide an image of an average doctor practicing in this country. Lack of correct answers does not always automatically translate to wrong clinical decisions in a real-life situation. There are 162000 licensed adult physicians and 65000 practicing paediatricians in Russia, which does not allow data extrapolation to the whole Russian medical fraternity. As the survey was presented in Russian, results cannot be generalised to medical professionals living in other countries. However, we believe that our results are closer to a best-case interpretation and therefore are all the more concerning.

Although limitations are clear, results of this survey suggest a commondeficit of knowledge on anaphylaxis diagnosis and management in Russia. Despite anaphylaxis guidelines being easily accessible via EAACI and RAACI websites they are not promoted well enough among Russian health professionals. Our data highlight the need for educational programme development and changes to the undergraduate and postgraduate medical education curriculum. This should lead to a better care quality for children with food allergy at risk of anaphylactic events and may potentially reduce morbidity and mortality. Furthermore, detection of existing gaps in

For peer review only

anaphylaxis diagnosis and management is important [16] at a time when food allergy prevalence is rising, particularly in the paediatric population.

Acknowledgments

We would like to thank physicians that kindly agreed to complete the survey and Vrachirf.ru website team for their incredible help with the survey.

Contributorship statement

DM, MT, IK and JW designed a questionnaire. AA and AP provided an independent evaluation of the survey instrument and assisted with the pre-survey pilot testing. DM and MT conceived the study, designed the methods and coordinated data collection. DM wrote the first draft of the paper. MT, JW contributed to the writing of the paper. All authors critically revised the manuscript and approved the final version.

Competing interests

None declared.

Funding

This research received no specific grant from any funding agency in the public, commercial, or not-for-profit sectors. However, JW is funded through the National Institute of Health Research (NIHR), Collaboration for Leadership in Applied Health Research and Care for NW London. The views expressed in the paper are those of the authors and are not necessarily those of the NHS, the NIHR or the Department of Health.

Author Data sharing statement

The Excel data set is available from corresponding author on request.

Figure 1 Physicians choice of adrenalin as a first line treatment of anaphylaxis. This graph shows proportion of physicians to choose: a) IM adrenaline 1:1000 or b) IM prednisolone as a first line treatment, depending on their choice of diagnosis.

References

1. National Institute for Health and Care Excellence (NICE). Food allergy. London: NICE, 2016.

For peer review only

2. Flokstra-de Blok BM, Dubois AE, Vlieg-Boerstra BJ, et al. Health-related quality of life of food allergic patients: comparison with the general population and other diseases. *Allergy* 2010;65(2):238-44. doi: 10.1111/j.1398-9995.2009.02121.x

3. Diwakar L, Cummins C, Ryan R, et al. Prescription rates of adrenaline auto-injectors for children in UK general practice: a retrospective cohort study. *Br J Gen Pract* 2017 doi: 10.3399/bjgp17X689917

4. Muraro A, Roberts G, Worm M, et al. Anaphylaxis: guidelines from the European Academy of Allergy and Clinical Immunology. *Allergy* 2014;69(8):1026-45. doi: 10.1111/all.12437

5. Panesar SS, Javad S, de Silva D, et al. The epidemiology of anaphylaxis in Europe: a systematic review. *Allergy* 2013;68(11):1353-61. doi: 10.1111/all.12272

6. Nwaru BI, Hickstein L, Panesar SS, et al. The epidemiology of food allergy in Europe: a systematic review and meta-analysis. *Allergy* 2014;69(1):62-75. doi: 10.1111/all.12305

7. Krugman SD, Chiaramonte DR, Matsui EC. Diagnosis and management of food-induced anaphylaxis: a national survey of pediatricians. *Pediatrics* 2006;118(3):e554-60. doi: 10.1542/peds.2005-2906

8. de Silva IL, Mehr SS, Tey D, et al. Paediatric anaphylaxis: a 5 year retrospective review. *Allergy* 2008;63(8):1071-6. doi: 10.1111/j.1398-9995.2008.01719.x

9. Hompes S, Kohli A, Nemat K, et al. Provoking allergens and treatment of anaphylaxis in children and adolescents--data from the anaphylaxis registry of German-speaking countries. *Pediatr Allergy Immunol* 2011;22(6):568-74. doi: 10.1111/j.1399-3038.2011.01154.x

10. Vetander M, Helander D, Flodstrom C, et al. Anaphylaxis and reactions to foods in children--a population-based case study of emergency department visits. *Clin Exp Allergy* 2012;42(4):568-77. doi: 10.1111/j.1365-2222.2011.03954.x

11. Simpson AB, Glutting J, Yousef E. Food allergy and asthma morbidity in children. *Pediatr Pulmonol* 2007;42(6):489-95. doi: 10.1002/ppul.20605

12. Physicians RCo. Why asthma still kills: the National Review of Asthma Deaths (NRAD) Confidential Enquiry report. . London: Royal College of Physicians, 2014.

13. Altman AM, Camargo CA, Jr., Simons FE, et al. Anaphylaxis in America: A national physician survey. *J Allergy Clin Immunol* 2015;135(3):830-3. doi: 10.1016/j.jaci.2014.10.049

14. Russian Association of Allergy and Clinical Immunology (RACI). Federal Clinical Recommendations on Anaphylactic Shock. 2013:1-13.

15. Russian Association of Allergy and Clinical Immunology (RACI). Federal Clinical Recommendations on Anaphylaxis diagnosis and management. 2015:1-18.

16. Wang J, Young MC, Nowak-Wegrzyn A. International survey of knowledge of food-induced anaphylaxis. *Pediatr Allergy Immunol* 2014;25(7):644-50. doi: 10.1111/pai.12284

17. Wang J, Sicherer SH, Nowak-Wegrzyn A. Primary care physicians' approach to food-induced anaphylaxis: a survey. *J Allergy Clin Immunol* 2004;114(3):689-91.

18. Grabenhenrich L, Hompes S, Gough H, et al. Implementation of anaphylaxis management guidelines: a register-based study. *PLoS One* 2012;7(5):e35778. doi: 10.1371/journal.pone.0035778

19. Grabenhenrich LB, Dolle S, Moneret-Vautrin A, et al. Anaphylaxis in children and adolescents: The European Anaphylaxis Registry. *J Allergy Clin Immunol* 2016;137(4):1128-37 e1. doi: 10.1016/j.jaci.2015.11.015

20. Tole JW, Lieberman P. Biphasic anaphylaxis: review of incidence, clinical predictors, and observation recommendations. *Immunol Allergy Clin North Am* 2007;27(2):309-26, viii. doi: 10.1016/j.iac.2007.03.011

For peer review only

21. Stapel SO, Asero R, Ballmer-Weber BK, et al. Testing for IgG4 against foods is not recommended as a diagnostic tool: EAACI Task Force Report. *Allergy* 2008;63(7):793-6. doi: 10.1111/j.1398-9995.2008.01705.x
22. Bock SA. AAAAI support of the EAACI Position Paper on IgG4. *J Allergy Clin Immunol* 2010;125(6):1410. doi: 10.1016/j.jaci.2010.03.013
23. Carr S, Chan E, Lavine E, et al. CSACI Position statement on the testing of food-specific IgG. *Allergy Asthma Clin Immunol* 2012;8(1):12. doi: 10.1186/1710-1492-8-12
24. De Schryver S, Halbrich M, Clarke A, et al. Tryptase levels in children presenting with anaphylaxis: Temporal trends and associated factors. *J Allergy Clin Immunol* 2016;137(4):1138-42. doi: 10.1016/j.jaci.2015.09.001
25. Nulty DD. The adequacy of response rates to online and paper surveys: what can be done? *Assessment & Evaluation in Higher Education* 2008;33(3):301–14.

For peer review only

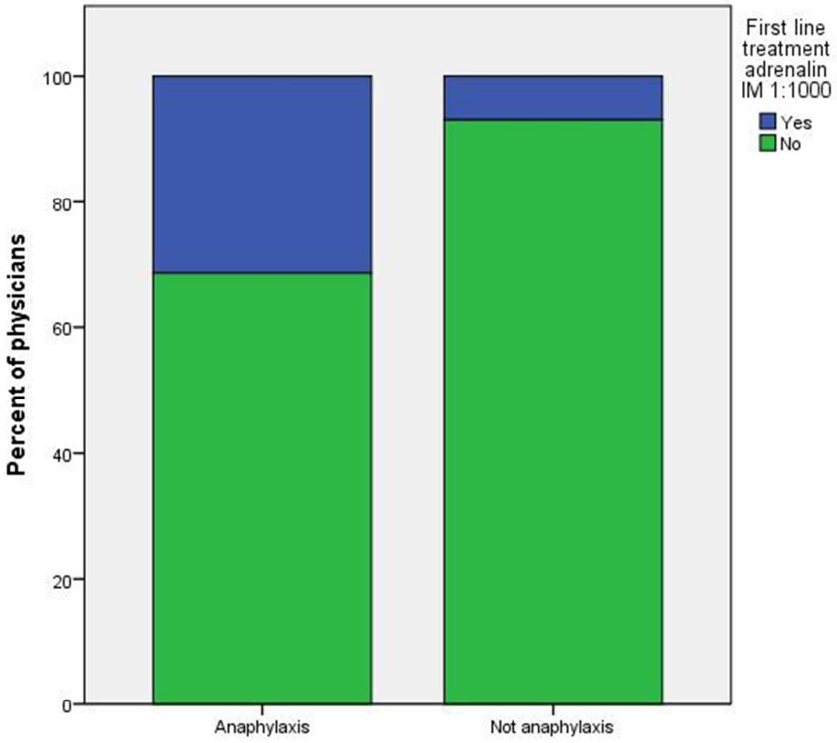

53x42mm (300 x 300 DPI)

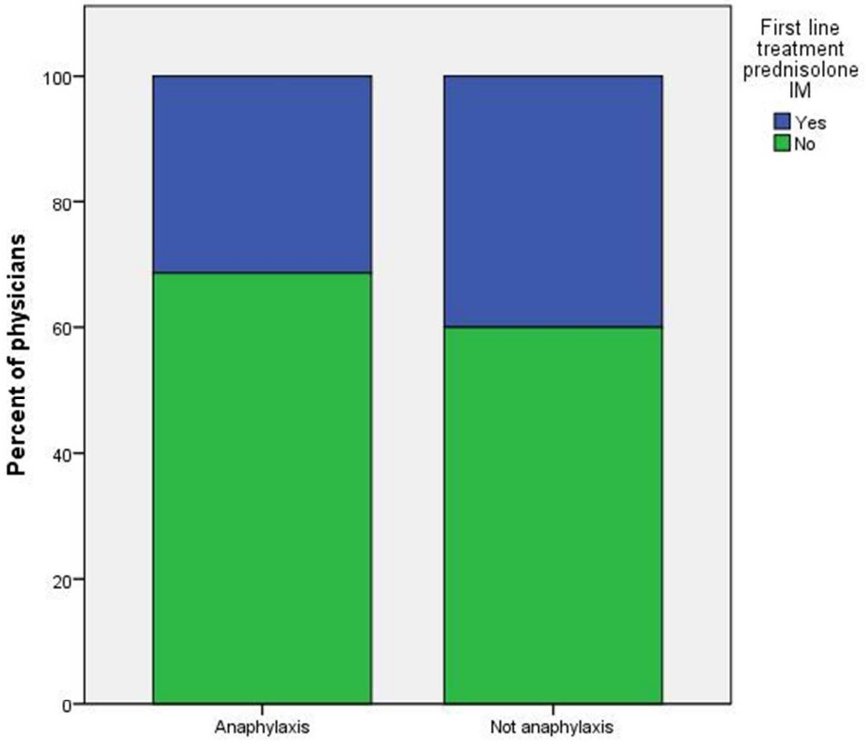

53x42mm (300 x 300 DPI)

Supplementary document 1

We would be very grateful for your participation in our study by completing this survey. It should take you approximately 10 minutes to complete. We will not collect any information that could identify you personally or the hospital where you work.

In this survey you will find three clinical scenarios and we would appreciate if you could address them according to your own clinical knowledge and expertise without seeking advice from other parties. It will help us to provide fair and objective evaluation of the results.

We hope to publish the results of the survey in 2016 and look forward to sharing these with you.

Sex:

- ☐ Male
- ☐ Female

Age: ___________

Country and city of residence:

Primary specialty:

Clinical practice settings:

A) primary care

B) primary practice

C) secondary care

D) tertiary care

Years in clinical practice:

- ☐ 1-2
- ☐ 3-5
- ☐ 6-10
- ☐ 11-15
- ☐ More than 15

Years in specialty practice (if applicable) ___________

For peer review only

Next page of the online questionnaire…

Clinical scenario 1:

During a busy weekend A&E shift, you are seeing a 6-year-old boy. His main complaint at admission is syncope. 10 minutes after eating Chinese meal at a friend's house he developed an urticarial rash on the neck. 5 minutes after the rash appearance he complained of abdominal cramping and vomited twice. After 15 minutes, he felt dizzy, developed a wheeze and fainted. He have been delivered to the hospital 35 minutes after occurrence of the first symptopms. It is not known if he has food allergy but he had 2 hospitalizations for asthma in the last year.

On examination, his heart rate is 90, blood pressure 90/45mmHg, respiratory rate 22, and oxygen saturation 94%. His weight is 22 kg. He has diffuse wheezing and is in moderate respiratory distress. He was not carrying his salbutamol inhaler and hasn't received any medications yet.

What is the most likely diagnosis?

___

Is any treatment required?

☐ Yes
☐ No

if yes, please choose the right answer/s (can be more than one option)

1) Oxygen
2) Salbutamol inhaler
3) Antihistamine (Cetirizine) orally
4) Antihistamine (Chlorphenamine) orally
5) Immediately start first line treatment - Antihistamine (Chlorphenamine) IV
6) Call PICU
7) Immediately start first line treatment - Adrenaline IM 1:10 000
8) Immediately start first line treatment - Adrenaline IM 1:1000
9) Metoclopramide
10) Adrenaline IV 1:100 if other treatment is unsuccessful
11) Immediately start first line treatment - Activated Charcoal
12) Inhaled Steroids
13) Oral prednisolone, in addition to first line treatment
14) Prednisolone IM as a first line treatment
15) Valproates
16) Midodrine
17) None of the above

if none of the above or any additional medications, please provide your options:

___

For peer review only

Next page of the online questionnaire…

On further questioning when the boy had recovered, he reported that the Chinese meal contained egg fried rice, beef, bean shoots, sesame, cashew nuts and peanuts. He has previously sometimes complained of itchy throat after eating chocolate and he had eczema in infancy worsened when eating egg. However, he has recently had egg without any reaction.

What tests would you do?

a) Full blood count
b) ECG
c) Echocardiogram
d) Urinalysis
e) Serum tryptase
f) Chest X-ray
g) EEG
h) sIgE or skin prick test to Egg
i) sIgE or skin prick test to Peanut, Cashew and Sesame
j) sIgG test to multiple food allergens
k) MRI
l) Spirometry
m) None of the above

if none of the above, or any additional tests, please provide your options:

____

____

What advice and further treatment would you give to this patient

____

____

For peer review only

Checklist from the https://www.equator-network.org/reporting-guidelines/good-practice-in-the-conduct-and-reporting-of-survey-research/ as recommended.

When reporting survey research, it is essential that a number of key points are covered (though the length and depth of reporting will be dependent upon journal style). These key points are presented as a 'checklist' below:

1. Explain the purpose or aim of the research, with the explicit identification of the research question.	✓
2. Explain why the research was necessary and place the study in context, drawing upon previous work in relevant fields (the literature review).	✓
3. Describe in (proportionate) detail how the research was done.	✓
a. State the chosen research method or methods, and justify why this method was chosen.	✓
b. Describe the research tool. If an existing tool is used, briefly state its psychometric properties and provide references to the original development work. If a new tool is used, you should include an entire section describing the steps undertaken to develop and test the tool, including results of psychometric testing.	✓
c. Describe how the sample was selected and how data were collected, including:	✓
iv.How were potential subjects identified?	✓
v.How many and what type of attempts were made to contact subjects?	✓
vi.Who approached potential subjects?	✓
vii.Where were potential subjects approached?	✓
viii.How was informed consent obtained?	✓
ix.How many agreed to participate?	✓
x.How did those who agreed differ from those who did not agree?<br><br>It is very hard to meet this recommendation due to a study design. Surveyed physicians voluntarily accessed the survey online and data available provides information and details only on those respondents who accessed the survey. We do not know how they differ from those physicians deciding not to undertake the survey.	✗
xi.What was the response rate?	✓
4. Describe and justify the methods and tests used for data analysis.	✓
5. Present the results of the research. The results section should be clear, factual, and concise.	✓
6. Interpret and discuss the findings. This 'discussion' section should not simply reiterate results; it should provide the author's critical reflection upon both the results and the processes of data collection. The discussion should assess how well the study met the research question, should describe the	✓

For peer review only - http://bmjopen.bmj.com/site/about/guidelines.xhtml

problems encountered in the research, and should honestly judge the limitations of the work.	
7. Present conclusions and recommendations.	✓

For peer review only

BMJ Open

A national survey of Russian physicians' knowledge of diagnosis and management of food induced anaphylaxis

Journal:	*BMJ Open*
Manuscript ID	bmjopen-2017-015901.R2
Article Type:	Research
Date Submitted by the Author:	27-Apr-2017
Complete List of Authors:	Munblit, Daniel; Pervyj Moskovskij gosudarstvennyj medicinskij universitet imeni I M Secenova, Faculty of Pediatrics; Imperial College London, Paediatrics<br>Treneva, Marina; Veltischev Clinical Pediatric Research Institute of Pirogov Russian National Research Medical University; International Inflammation (in-FLAME) Network of the World Universities Network<br>Korsunskiy, Ilya; Pervyj Moskovskij gosudarstvennyj medicinskij universitet imeni I M Secenova, Faculty of Pediatrics<br>Asmanov, Alan; Veltischev Clinical Pediatric Research Institute of Pirogov Russian National Research Medical University<br>Pampura, Alexander; Veltischev Clinical Pediatric Research Institute of Pirogov Russian National Research Medical University; International Inflammation (in-FLAME) Network of the World Universities Network<br>Warner, John O.; Imperial College London, Paediatrics; International Inflammation (in-FLAME) Network of the World Universities Network
**Primary Subject Heading**:	Immunology (including allergy)
Secondary Subject Heading:	Paediatrics
Keywords:	anaphylaxis, survey, food-induced anaphylaxis, anaphylaxis knowledge

SCHOLARONE™
Manuscripts

A national survey of Russian physicians' knowledge of diagnosis and management of food induced anaphylaxis

Daniel Munblit [1,2,4*], Marina Treneva [3,4], Ilya Korsunskiy [2], Alan Asmanov [3], Alexander Pampura [3,4], John O Warner [1,4,5]

[1] Department of Paediatrics, Imperial College London, UK
[2] Faculty of Pediatrics, I. M. Sechenov First Moscow State Medical University, Russia
[3] Allergy Department, Veltischev Clinical Pediatric Research Institute of Pirogov Russian National Research Medical University, Russia
[4] International Inflammation (in-FLAME) Network of the World Universities Network
[5] National Institute of Health Research Collaboration for Leadership in Applied Health Research and Care for NW London, UK

***Corresponding author:**
Dr. Daniel Munblit
Wright Fleming Building
Norfolk Place
London W2 1PG
Tel. +447898257151
Email: daniel.munblit08@imperial.ac.uk

Key words: anaphylaxis, survey, food-induced anaphylaxis, anaphylaxis knowledge

Running Title: Physicians knowledge of diagnosis and management of food induced anaphylaxis in Russia

Funding Source: The authors have no funding relationships relevant to the article to disclose.

Financial Disclosure: The authors have no financial relationships relevant to the article to disclose.

Conflict of Interest: The authors have no conflicts of interest to disclose.

Word count: 2722

Abstract

Objectives: Food allergy is an increasing burden worldwide and is a common problem within paediatric populations, affecting 5-8% of children. Anaphylaxis caused by food proteins is a potentially life-threatening condition and all healthcare practitioners should be aware of its recognition and management. Russia is the largest country in Europe but it is still unknown whether physicians are prepared to diagnose and manage food induced anaphylaxis effectively. We aimed to examine physicians' knowledge of diagnosis and management of food induced anaphylaxis.

For peer review only

Setting, population and outcomes: A survey was designed and published online at VrachiRF.ru website (for registered Russian-speaking practicing physicians). We obtained information on respondents' clinical settings, experience and specialty. Survey questions were based upon a characteristic clinical scenario of anaphylaxis due to food ingestion. Outcome measures consisted of correct answers to the anaphylaxis diagnosis and management questions.

Results: From a total of 707 of physicians accessed in the survey, 315 (45%) responded to the clinical scenario. 16 respondents reported training in allergy-immunology and have been excluded from the analysis, leaving the final sample size of 299. Respondents were paediatricians (68%) and other specialties adult physicians (32%). Overall, 100 (33%) of respondents diagnosed anaphylaxis but only 29% of those making the correct diagnosis administered adrenalin (1:1000) IM. Respondents working in secondary/tertiary clinics diagnosed anaphylaxis significantly more often (p=0.04) when compared to primary care/private practice physicians. This difference was also apparent as the most important influence on responses in the multivariate analysis.

Conclusions: In this national sample of Russian physicians we found poor knowledge in both, anaphylaxis diagnosis and management. Our data shows that the chance of being properly diagnosed with anaphylaxis is 33% and being appropriately treated with adrenalin is 10% respectively. These findings highlight lack of anaphylaxis knowledge amongst Russian physicians, both paediatricians and other specialists and illustrates the urgent need for allergy/anaphylaxis training.

Strengths and limitations of this study

- Strengths of this survey include the large number of respondents from different medical backgrounds. This is the first survey of anaphylaxis knowledge in Russian physicians and provides an insight into current ability of Russian doctors to diagnose and manage anaphylaxis. As all surveyed physicians answered the same set of questions it allows for comparisons of their knowledge between the groups.
- Limitations of the study are the use of non-standardised and validated survey instruments. The distribution via VrachiRF.ru website where respondents may be more educated than average physicians, and the 45% response rate probably present a best rather than worst case scenario.

For peer review only

Introduction

Allergic conditions are a major problem worldwide and their prevalence have increased considerably in the recent decades with a quarter of school-age children in Europe being allergic. Among allergic diseases, food allergy (FA) has become a significant burden with 6-8% of children under the age of three having allergy to at least one food [1]. FA has detrimental effects on the quality of life [2] and health/economics with prescribing of adrenalin auto-injectors (AAIs) having risen over 350 fold in the UK from 2000 to 2012 [3].

Anaphylaxis is a 'severe, life-threatening generalised systemic hypersensitivity reaction' [4]. Details on anaphylaxis prevalence, the main triggers and mortality rates are available in some countries, but missing in many geographical locations. A recent meta-analysis undertaken by the European Academy of Allergy and Clinical Immunology (EAACI) provided an estimated pooled prevalence of anaphylaxis of 0.3% [5]. No data on anaphylaxis prevalence and/or mortality rates are available from the Russian Federation. The reasons for the lack of research in this field in Russia remain unclear but may be partially explained by lack of a unified digital registry of anaphylaxis events and unavailability of AAIs, as none of the existing brands are registered in Russian Federation. The registration is a prerequisite for a drug to be approved for clinical use. The process of approval for medical use of a drug includes drug quality, efficacy, safety and, in case with autoinjectors, delivery, assessment by an authorised state body.

Food allergy is a major health concern and is increasing in prevalence reaching an overall pooled point prevalence of 0.9% when confirmed by oral food challenge [6]. Data from the United States shows that food induced anaphylaxis is a cause of 2000 hospitalisations and 200 death a year [7]. Food is the leading cause of anaphylactic reactions, especially in paediatric populations [8 9] and when associated with co-existing asthma has an increased risk of death[10]. Coexistence of food allergy and asthma is not an unusual event as 4-8% of asthmatic children with asthma have food allergy, and more than a third of children with food allergy have asthma [11]. The most recent UK-wide investigation on 195 asthma deaths found that many people died of asthma with co-existent allergy between 2012 and 2013 [12].

EAACI has underlined the importance of anaphylaxis recognition as a clinical emergency, and highlighted the need for healthcare professionals to be aware of it's diagnosis and management [4]. The EAACI Taskforce on anaphylaxis drafted guidelines covering all aspects of recognition,

For peer review only

risk factor assessment, and the management of patients. Despite these efforts, levels of anaphylaxis recognition and management knowledge is still far from perfect [7 13].

The Russian Association of Allergy and Clinical Immunology (RAACI) local guidelines provide physicians with the basic principles of anaphylaxis diagnosis and management [14 15] but the degree of anaphylaxis understanding amongst Russian physicians is unknown. Our survey aimed to provide information of Russian physicians knowledge of anaphylaxis diagnosis and management.

Methods

Study design and population

A cross-sectional study was conducted in January 2016 using an online survey published on the VrachiRF (vrachirf.ru) website. VrachiRF is a popular Russian professional website for registered physicians (with about half a million subscribers) which provides clinically relevant information and latest news in the medical field. The website sends daily emails to all the members of VrachiRF community with the latest medical news and updates and a special email on Sunday, providing members with the top news of the week. VrachiRF placed a survey on the website for 4 weeks (between February and March 2016), responses were collected and anonymised data presented in an Excel spreadsheet. The study population included paediatricians and adult physicians of various specialities. The survey was conducted following ethical approval by Moscow Clinical Hospital №9, named after G.N.Speransky, Ethics committee.

Survey questionnaire

We developed a case based survey in Russian to evaluate physicians knowledge of anaphylaxis diagnosis and management. The survey consisted of a clinical scenario; open-ended questions on diagnosis; multiple choice questions on treatment, required clinical tests and recommendations. The clinical scenario described an anaphylactic event with multiple system involvement:

During a busy weekend A&E shift, you are seeing a 6-year-old boy. His main complaint at admission is syncope. 10 minutes after eating a Chinese meal at a friend's house he developed an urticarial rash on the neck. 5 minutes after the rash appearance he complained of abdominal cramping and vomited twice. After 15 minutes, he felt dizzy, developed a wheeze and fainted.

For peer review only

He was delivered to the hospital 35 minutes after occurrence of the first symptoms. It is not known if he has food allergy but he had 2 hospitalizations for asthma in the last year.
On examination, his heart rate is 90, blood pressure 90/45mmHg, respiratory rate 22, and oxygen saturation 94%. His weight is 22 kg. He has diffuse wheezing and is in moderate respiratory distress. He was not carrying his salbutamol inhaler and hasn't received any medications yet.

As no validated survey instrument exists the development of this questionnaire was based on allergy expert opinion and previously published data. The survey was developed by the research team, consisting of paediatricians and allergy specialists. It was pilot tested with external adult physicians, paediatricians and allergy specialists for clarity, prior to its use.

The first question asked responders to provide the most likely diagnosis for the clinical scenario in an open-answer form. The next page of online survey provided respondents with additional information on the patient: *"on further questioning when the boy had recovered, he reported that the Chinese meal contained egg fried rice, beef, bean shoots, sesame, cashew nuts and peanuts. He has previously sometimes complained of itchy throat after eating chocolate and he had eczema in infancy worsened when eating egg. However, he has recently had egg without any reaction.* "The following questions focused on patient management and additional investigations required which were presented in the form of a multiple choice. Additional information on participants included age, gender, years of clinical experience, speciality, country and city of residence, clinical practice settings. Full version of the survey is available as a supplementary document 1.

Statistical analysis

Data were imported from the survey website into Microsoft Excel 2013, cleaned to detect any missing or invalid variables and then converted into the SPSS database. Statistical analysis was performed using IBM SPSS Statistics for Windows, Version 22.0 (Armonk, NY: IBM Corp.).

Statistical significance between the groups have been assessed using x^2 for categorical variables and Mann – Whitney U test for continuous variables. In order to evaluate any association between the demographic factors and ability to make the right diagnosis and treatment, logistic

For peer review only

regression was used. Results were considered significant when p-values were reported at a level less than 0.05.

Results

Characteristics of the study population

The study was conducted using an online survey questionnaire tool. Of the 707 physicians accessed the survey, 315 (45%) responded to the clinical scenario and answered all the questions. 16 respondents reported that they had received training in allergy-immunology and have been excluded from the analysis, as we were not aiming to survey specialists, leaving the final sample size of 299.

The study population were predominantly women (85%), in their fifties (mean age of 47.7 years), with two thirds (68%) of respondents worked in the paediatric field. More than half (57%) of the surveyed physicians work in primary care settings. 70% of the participants had practiced for more than 15 years. The demographic characteristics of study participants and physicians decided not to answer the survey are summarized in Table 1.

Characteristic	N (%) Respondents	N (%) Non-respondents	P value (χ2)
**Gender**			
**Male**	44 (15%)	65 (17%)	
**Female**	255 (85%)	325 (83%)	0.48
**Age**	47.7 (10.7)ǂ	49.0 (9.44)ǂ	0.06
**Specialty**			
**Paediatrics**	203 (68%)	205 (53%)	
**Other**	96 (32%)	185 (47%)	**<0.01**
**Clinical settings**			
**Primary care**	169 (57%)	224 (57%)	
**Secondary care**	67 (22%)	94 (24%)	
**Tertiary care**	36 (12%)	42 (11%)	
**Private practice only**	27 (9%)	32 (8%)	0.92
**Clinical experience**			
**1-5 years**	29 (10%)	25 (6%)	
**6-10 years**	31 (10%)	33 (9%)	
**11-15 years**	30 (10%)	54 (14%)	
**More than 15 years**	209 (70%)	278 (71%)	0.17

Table 1 Characteristics of the survey respondents and non-respondents. ǂ - mean (SD)

For peer review only

Respondents knowledge of anaphylaxis diagnosis and management

Thirty three percent of surveyed physicians diagnosed the presented patient with anaphylaxis. The most popular choice for first line treatment options was IM prednisolone (37%) followed by chloropyramine (26%), with only 15% of respondents suggested adrenalin (1:1000) IM as a treatment of choice. Of those making the correct diagnosis only 29% selected adrenalin as a first-line treatment (Figure 1). Forty six percent of surveyed physicians considered salbutamol use as appropriate and 47% selected supplemental oxygen as a part of the necessary treatment. Data on respondents knowledge is presented in Table 2.

**Outcomes**	**Responses**	**N=299**<br>**N (%)**
Diagnosis		
**What is the most likely diagnosis?**	Anaphylaxis	99 (33)
	Not anaphylaxis	200 (67)
Treatment		
**Use adrenalin 1:1000 IM as a first line treatment**	Yes	45 (15)
	No	254 (85)
**Use adrenalin 1:10000 IM as a first line treatment**	Yes	31 (10)
	No	268 (90)
**Use chloropyramine as a first line treatment**	Yes	79 (26)
	No	220 (74)
**Use prednisolone IM as a first line treatment**	Yes	111 (37)
	No	188 (63)
**Uses prednisolone IM in addition to other therapy**	Yes	109 (37)
	No	190 (63)
**Oxygen supply**	Yes	141 (47)
	No	158 (53)
**Salbutamol inhaler**	Yes	139 (46)
	No	160 (54)
**Antihistamine (cetirizine)**	Yes	11 (4)
	No	288 (96)
**Inhaled steroids**	Yes	110 (37)
	No	189 (63)
Further investigations needed		
**Serum tryptase**	Yes	69 (23)
	No	230 (77)
**Spirometry**	Yes	199 (67)
	No	100 (33)
**Chest X-ray**	Yes	144 (48)
	No	155 (52)
**SPT or sIgE testing to food allergens**	Yes	213 (71)
	No	86 (29)
**sIgG testing to food allergens**	Yes	187 (63)
	No	112 (37)
**Stool ova and parasite exam**	Yes	96 (32)
	No	203 (78)

Table 2 Respondents answers on anaphylaxis Clinical scenario.

For peer review only

48 and 67% selected chest X-ray and spirometry respectively, as required investigations. Most of the respondents considered sIgE (71%) and sIgG (63%) to food allergens testing as necessary tests but only 23% suggested serum tryptase measurement

Respondents working in secondary/tertiary clinics diagnosed patient with anaphylaxis significantly more often (p=0.04) when compared to primary care/private practice physicians. This was also the most important factor in the regression model, upon adjustment for participants sex, age, specialty and clinical experience. Neither clinical experience, nor specialty significantly influenced participants decision to use adrenalin. The medication choice was not associated with the participant characteristics (Table 3).

Multivariate analysis outcomes (Table 4) confirmed univariate analysis results showing that clinical setting is the most important factor influencing physicians ability to diagnose anaphylaxis. Those working in primary care/private practice make the correct diagnosis less often than in secondary/tertiary care hospitals OR 0.51 (95% CI 0.30 – 0.86). Participants diagnosing anaphylaxis were much more likely to treat patient with adrenalin OR 5.65 (95% CI 2.81 – 11.36). Prednisolone use was independent of participant characteristics. Two factors influencing chloropyramine prescription were age (younger age decreased the likelihood of prescribing) OR 0.97 (95% CI 0.93 – 0.99) and ability to diagnose anaphylaxis (right diagnosis decreased the likelihood of prescribing) OR 0.35 (95% CI 0.19 – 0.67), respectively.

For peer review only

Respondents characteristic	Diagnosed patient with anaphylaxis, %	OR (95% CI)	P value	Used adrenalin 1:1000 IM, %	OR (95% CI)	P value	Used prednisolone IM, %	OR (95% CI)	P value	Used chloropyramine, %	OR (95% CI)	P value
Clinical settings												
Primary care/Private practice	29	0.59 (0.36 - 0.98)	**0.04**	13	0.68 (0.35-1.29)	0.23	41	1.60 (0.96-2.66)	0.07	27	1.18 (0.69-2.05	0.54
Secondary and/or Tertiary care	41			18			30			24		
Clinical experience												
1-10 years	32	0.92 (0.50-1.69)	0.79	15	0.99 (0.45-2.20)	0.99	38	1.07 (0.60-1.91)	0.83	28	1.13 (0.60-2.12)	0.71
>10 years	34			15			37			26		
Specialty												
Paediatrics	34	1.22 (0.72-2.05)	0.46	16	1.36 (0.67-2.77)	0.40	39	1.36 (0.82-2.28)	0.23	29	1.43 (0.81-2.53)	0.22
Other specialities	30			13			32			22		

Table 3 Diagnosis and first line treatment amongst different respondent groups. x^2 test is used for the group comparison. Statistically significant difference (p<0.05) appear in bold.

	Anaphylaxis diagnosis Odds Ratio (95% CI)	P Value	IM Adrenalin Odds Ratio (95% CI)	P Value	Prednisolone Odds Ratio (95% CI)	P Value	Chloropyramine Odds Ratio (95% CI)	P Value
Sex	0.75 (0.36 – 1.57)	0.45	0.71 (0.24 – 2.10)	0.54	1.69 (0.85 – 3.83)	0.14	1.49 (0.70 – 3.15)	0.30
Age	0.97 (0.95 – 1.00)	0.10	0.98 (0.94 – 1.03)	0.42	1.00 (0.98 – 1.04)	0.61	0.97 (0.93 – 0.99)	**0.04**
Specialty	1.34 (0.77 – 2.35)	0.31	1.30 (0.60 – 2.83)	0.51	1.43 (0.82 – 2.50)	0.20	1.59 (0.85 – 2.97)	0.15
Clinical settings	0.51 (0.30 – 0.86)	**0.01**	0.74 (0.36 – 1.50)	0.40	1.55 (0.90 – 2.68)	0.11	0.95 (0.52 – 1.73)	0.86
Clinical experience	1.33 (0.59 – 2.99)	0.49	1.39 (0.45 – 4.31)	0.57	0.94 (0.43 – 2.05)	0.88	1.93 (0.81 – 4.61)	[illegible]
Anaphylaxis diagnosis	NA	NA	5.65 (2.81 – 11.36)	**<0.01**	0.73 (0.44 – 1.24)	0.24	0.35 (0.19 – 0.67)	**<0.01**

Table 4 Multivariate predictive model for factors influencing physicians decision on anaphylaxis diagnosis and first line management choice. Statistically significant difference (p<0.05) appear in bold.

For peer review only

Discussion

Anaphylaxis awareness has received more attention over the last decade. The findings of our study provide the first insight into knowledge of anaphylaxis diagnosis and management in Russia. Although recent reports from several countries [16] suggest that lack of knowledge in anaphylaxis diagnosis and management is not "as great as previously published", differing patterns are seen in Russia. Sixty seven percent of respondents did not recognise anaphylaxis in a very clearly presented clinical scenario, for which anaphylaxis should be strongly considered. Approximately one in ten professionals made an appropriate choice of adrenalin as a first line treatment and only a third of those who rightfully diagnosed anaphylaxis provided appropriate medication. Given a response rate of 45%, our results are more likely to represent a best rather than worst case scenario. It suggests that food allergic patients could be at increased risk of fatal reaction due to anaphylaxis in Russia and this danger is even higher considering lack of availability of AAIs in Russia.

Overall, only ten percent of surveyed physicians were able to both diagnose and provide appropriate treatment for a child with food-induced anaphylaxis, when assessed using a clinical scenario survey instrument. These numbers are much lower than in any of the previous studies up to date [7 16 17], but corresponds well with real-life data, showing that anaphylaxis management is still far from satisfactory based on established recommendations. Analysis of a large anaphylaxis registry showed discrepancies between existing management guidelines and their implementation in clinical practice [18]. Similar outcomes have been recently confirmed from the European Anaphylaxis Registry database [19].

Two studies from US included anaphylaxis scenarios with no skin rashes present [7 13] as it is known that anaphylaxis can often manifest with no obvious cutaneous reaction. In our survey, we did not overcomplicate the clinical scenario, providing respondents with a combination of the cutaneous, cardiorespiratory, GI and generalized symptoms. While the majority of the physicians recognised an allergic reaction in the clinical scenario, only 33% of respondents diagnosed the patient as having anaphylaxis. This outcome shows that most of the doctors are not be aware of the anaphylaxis diagnostic criteria. Our clinical scenario clearly meets anaphylaxis diagnostic criteria stated within EAACI [4] and RAACI [14 15] guidelines. Poor EAACI guidelines awareness in Russia may be a result of a generally low level of English

For peer review only

comprehension but unawareness of RAACI guidelines is likely related to poor distribution and promotion.

Anaphylaxis recognition is the most important prerequisite for successful management, as physicians are more likely to administer adrenalin if they have correctly diagnosed the patient, as reported previously [7]. Our data is an agreement with the outcomes of previous studies. Among the factors potentially impacting participants knowledge we did not find any significant influence of primary specialty and/or clinical experience on physicians ability to diagnose anaphylaxis. Respondents working in the secondary or tertiary centres possess better knowledge on anaphylaxis diagnosis which may be explained by their daily exposure to highly specialised medical environment and involvement in academic activities.

Conversely chloropyramine use as a first line treatment was inversely associated with anaphylaxis diagnosis. To what extent this is due to resort to medications which are freely available rather than a lack of knowledge cannot be determined but the latter is more likely given the poor responses to further investigation questions.

Intramuscular adrenalin is the drug of choice in the scenario of an anaphylactic event and should be injected as soon as possible [18 19]. It is evident that in our survey, most of the respondents preferred intramuscular prednisolone or oral chloropyramine use, irrespective of their speciality, clinical experience and/or clinical settings. Prednisolone or other corticosteroids can be used for biphasic anaphylaxis prevention, which accounts for around 20% of anaphylactic cases [20], and may reduce the risk of late-phase respiratory symptoms [4], but it does not resolve acute anaphylaxis. First generation antihistamines are not an optimal choice for anaphylaxis management, especially when patient is lethargic, as they may cause drowsiness and mask respiratory symptoms [14].

Beta-2 agonists can be used during anaphylaxis to assist in the event of bronchoconstriction and the patients should be provided with supplemental oxygen [4] but only half of respondents selected these options as a part of required management.

The majority of respondents choose SPT/sIgE and/or sIgG testing to food allergens, to detect a possible trigger of reaction. The number of physicians prioritising SPT/sIgE over sIgG testing to

For peer review only

food was little different. These results suggest lack of differentiation between sIgE and sIgG amongst Russian physicians. Recently, a number of position papers has been released, warning practitioners against IgG testing use in the diagnosis of food allergy [21-23] but these papers are not easily available in Russia and no relevant statements has been provided by RAACI. Approximately one in four respondents considered serum tryptase as a useful test in the case scenario. Routine evaluation of serum tryptase levels is still a grey area but recent data suggests that it is particularly useful in severe anaphylactic events due to drugs and wasp/bee venom reactions. However, tryptase is not be raised in many cases of food induced anaphylaxis other than if milk is a causative allergen [24].

There were several limitations to the study. The survey is not standardised and validated. It was distributed via VrachiRF website which can be considered as one of the most popular sources for Russian physicians. Low response rate is a recognised issue in survey research, which potentially may lead to selection bias. Of the physicians accessed the survey 45% completed it, which is not ideal but slightly above average for online surveys [25]. It is not known how many doctors out of half a million subscribers accessed any part of the website during the month that the questionnaire was active. Respondents tended to work in paediatric field more often than non-respondents, probably due to the case scenario describing anaphylaxis in a child.

Clinicians using medical websites and responding to a competence questionnaire may possess better knowledge on a wide range of medical topics, meaning that survey outcomes do not necessarily provide an image of an average doctor practicing in this country. Lack of correct answers does not always automatically translate to wrong clinical decisions in a real-life situation. There are 162000 licensed adult physicians and 65000 practicing paediatricians in Russia, which does not allow data extrapolation to the whole Russian medical fraternity. As the survey was presented in Russian, results cannot be generalised to medical professionals living in other countries. However, we believe that our results are closer to a best-case interpretation and therefore are even more concerning.

Although limitations are clear, results of this survey suggest a common deficit of knowledge on anaphylaxis diagnosis and management in Russia. Despite anaphylaxis guidelines being easily accessible via EAACI and RAACI websites they are not promoted well enough among Russian health professionals. Our data highlight the need for educational programme development and

For peer review only

changes to the undergraduate and postgraduate medical education curriculum. This should lead to a better care quality for children with food allergy at risk of anaphylactic events and may potentially reduce morbidity and mortality. Furthermore, detection of existing gaps in anaphylaxis diagnosis and management is important [16] at a time when food allergy prevalence is rising, particularly in the paediatric population.

Acknowledgments

We would like to thank physicians that kindly agreed to complete the survey and Vrachirf.ru website team for their incredible help with the survey.

Contributorship statement

DM, MT, IK and JW designed a questionnaire. AA and AP provided an independent evaluation of the survey instrument and assisted with the pre-survey pilot testing. DM and MT conceived the study, designed the methods and coordinated data collection. DM wrote the first draft of the paper. MT, JW contributed to the writing of the paper. All authors critically revised the manuscript and approved the final version.

Competing interests

None declared.

Funding

This research received no specific grant from any funding agency in the public, commercial, or not-for-profit sectors. However, JW is funded through the National Institute of Health Research (NIHR), Collaboration for Leadership in Applied Health Research and Care for NW London. The views expressed in the paper are those of the authors and are not necessarily those of the NHS, the NIHR or the Department of Health.

Author Data sharing statement

The Excel data set is available from corresponding author on request.

Figure 1 Physicians choice of adrenalin as a first line treatment of anaphylaxis. This graph shows proportion of physicians to choose: a) IM adrenaline 1:1000 or b) IM prednisolone as a first line treatment, depending on their choice of diagnosis.

For peer review only

References

1. National Institute for Health and Care Excellence (NICE). Food allergy. London: NICE, 2016.
2. Flokstra-de Blok BM, Dubois AE, Vlieg-Boerstra BJ, et al. Health-related quality of life of food allergic patients: comparison with the general population and other diseases. *Allergy* 2010;65(2):238-44. doi: 10.1111/j.1398-9995.2009.02121.x
3. Diwakar L, Cummins C, Ryan R, et al. Prescription rates of adrenaline auto-injectors for children in UK general practice: a retrospective cohort study. *Br J Gen Pract* 2017 doi: 10.3399/bjgp17X689917
4. Muraro A, Roberts G, Worm M, et al. Anaphylaxis: guidelines from the European Academy of Allergy and Clinical Immunology. *Allergy* 2014;69(8):1026-45. doi: 10.1111/all.12437
5. Panesar SS, Javad S, de Silva D, et al. The epidemiology of anaphylaxis in Europe: a systematic review. *Allergy* 2013;68(11):1353-61. doi: 10.1111/all.12272
6. Nwaru BI, Hickstein L, Panesar SS, et al. The epidemiology of food allergy in Europe: a systematic review and meta-analysis. *Allergy* 2014;69(1):62-75. doi: 10.1111/all.12305
7. Krugman SD, Chiaramonte DR, Matsui EC. Diagnosis and management of food-induced anaphylaxis: a national survey of pediatricians. *Pediatrics* 2006;118(3):e554-60. doi: 10.1542/peds.2005-2906
8. de Silva IL, Mehr SS, Tey D, et al. Paediatric anaphylaxis: a 5 year retrospective review. *Allergy* 2008;63(8):1071-6. doi: 10.1111/j.1398-9995.2008.01719.x
9. Hompes S, Kohli A, Nemat K, et al. Provoking allergens and treatment of anaphylaxis in children and adolescents--data from the anaphylaxis registry of German-speaking countries. *Pediatr Allergy Immunol* 2011;22(6):568-74. doi: 10.1111/j.1399-3038.2011.01154.x
10. Vetander M, Helander D, Flodstrom C, et al. Anaphylaxis and reactions to foods in children--a population-based case study of emergency department visits. *Clin Exp Allergy* 2012;42(4):568-77. doi: 10.1111/j.1365-2222.2011.03954.x
11. Simpson AB, Glutting J, Yousef E. Food allergy and asthma morbidity in children. *Pediatr Pulmonol* 2007;42(6):489-95. doi: 10.1002/ppul.20605
12. Physicians RCo. Why asthma still kills: the National Review of Asthma Deaths (NRAD) Confidential Enquiry report. . London: Royal College of Physicians, 2014.
13. Altman AM, Camargo CA, Jr., Simons FE, et al. Anaphylaxis in America: A national physician survey. *J Allergy Clin Immunol* 2015;135(3):830-3. doi: 10.1016/j.jaci.2014.10.049
14. Russian Association of Allergy and Clinical Immunology (RACI). Federal Clinical Recommendations on Anaphylactic Shock. 2013:1-13.
15. Russian Association of Allergy and Clinical Immunology (RACI). Federal Clinical Recommendations on Anaphylaxis diagnosis and management. 2015:1-18.
16. Wang J, Young MC, Nowak-Wegrzyn A. International survey of knowledge of food-induced anaphylaxis. *Pediatr Allergy Immunol* 2014;25(7):644-50. doi: 10.1111/pai.12284
17. Wang J, Sicherer SH, Nowak-Wegrzyn A. Primary care physicians' approach to food-induced anaphylaxis: a survey. *J Allergy Clin Immunol* 2004;114(3):689-91.
18. Grabenhenrich L, Hompes S, Gough H, et al. Implementation of anaphylaxis management guidelines: a register-based study. *PLoS One* 2012;7(5):e35778. doi: 10.1371/journal.pone.0035778
19. Grabenhenrich LB, Dolle S, Moneret-Vautrin A, et al. Anaphylaxis in children and adolescents: The European Anaphylaxis Registry. *J Allergy Clin Immunol* 2016;137(4):1128-37 e1. doi: 10.1016/j.jaci.2015.11.015

For peer review only - http://bmjopen.bmj.com/site/about/guidelines.xhtml

20. Tole JW, Lieberman P. Biphasic anaphylaxis: review of incidence, clinical predictors, and observation recommendations. *Immunol Allergy Clin North Am* 2007;27(2):309-26, viii. doi: 10.1016/j.iac.2007.03.011
21. Stapel SO, Asero R, Ballmer-Weber BK, et al. Testing for IgG4 against foods is not recommended as a diagnostic tool: EAACI Task Force Report. *Allergy* 2008;63(7):793-6. doi: 10.1111/j.1398-9995.2008.01705.x
22. Bock SA. AAAAI support of the EAACI Position Paper on IgG4. *J Allergy Clin Immunol* 2010;125(6):1410. doi: 10.1016/j.jaci.2010.03.013
23. Carr S, Chan E, Lavine E, et al. CSACI Position statement on the testing of food-specific IgG. *Allergy Asthma Clin Immunol* 2012;8(1):12. doi: 10.1186/1710-1492-8-12
24. De Schryver S, Halbrich M, Clarke A, et al. Tryptase levels in children presenting with anaphylaxis: Temporal trends and associated factors. *J Allergy Clin Immunol* 2016;137(4):1138-42. doi: 10.1016/j.jaci.2015.09.001
25. Nulty DD. The adequacy of response rates to online and paper surveys: what can be done? *Assessment & Evaluation in Higher Education* 2008;33(3):301–14.

For peer review only

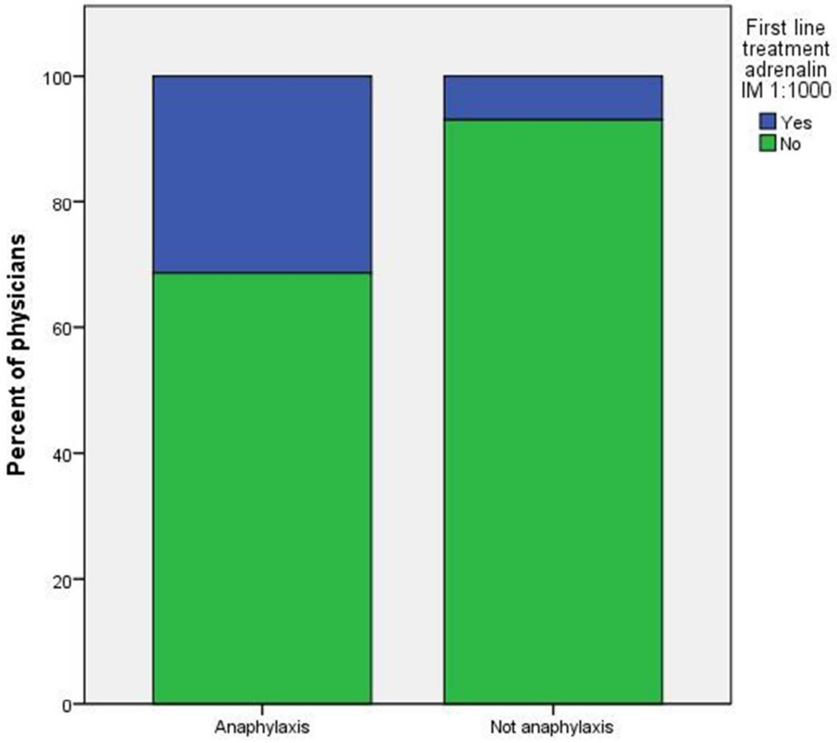

53x42mm (300 x 300 DPI)

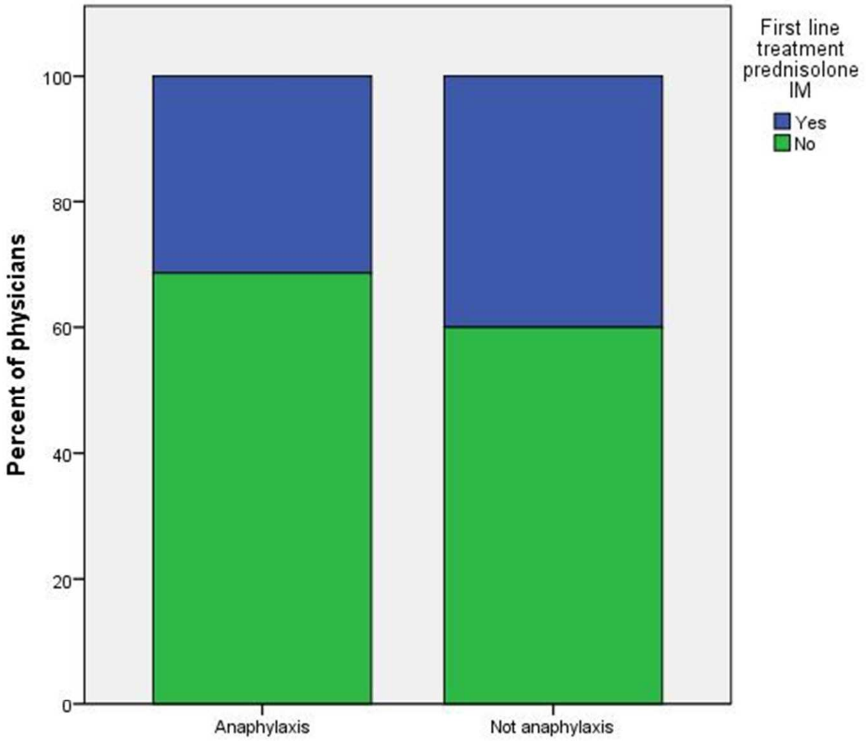

53x42mm (300 x 300 DPI)

For peer review only

Supplementary document 1

We would be very grateful for your participation in our study by completing this survey. It should take you approximately 10 minutes to complete. We will not collect any information that could identify you personally or the hospital where you work.

In this survey you will find three clinical scenarios and we would appreciate if you could address them according to your own clinical knowledge and expertise without seeking advice from other parties. It will help us to provide fair and objective evaluation of the results.

We hope to publish the results of the survey in 2016 and look forward to sharing these with you.

Sex:

- ☐ **Male**
- ☐ **Female**

Age: ___________

Country and city of residence:

Primary specialty:

Clinical practice settings:

A) primary care

B) primary practice

C) secondary care

D) tertiary care

Years in clinical practice:

- ☐ **1-2**
- ☐ **3-5**
- ☐ **6-10**
- ☐ **11-15**
- ☐ **More than 15**

Years in specialty practice (if applicable) ___________

For peer review only

Next page of the online questionnaire…

Clinical scenario 1:

During a busy weekend A&E shift, you are seeing a 6-year-old boy. His main complaint at admission is syncope. 10 minutes after eating Chinese meal at a friend's house he developed an urticarial rash on the neck. 5 minutes after the rash appearance he complained of abdominal cramping and vomited twice. After 15 minutes, he felt dizzy, developed a wheeze and fainted. He have been delivered to the hospital 35 minutes after occurrence of the first symptopms. It is not known if he has food allergy but he had 2 hospitalizations for asthma in the last year.

On examination, his heart rate is 90, blood pressure 90/45mmHg, respiratory rate 22, and oxygen saturation 94%. His weight is 22 kg. He has diffuse wheezing and is in moderate respiratory distress. He was not carrying his salbutamol inhaler and hasn't received any medications yet.

What is the most likely diagnosis?

____

Is any treatment required?

☐ Yes
☐ No

if yes, please choose the right answer/s (can be more than one option)

1) Oxygen
2) Salbutamol inhaler
3) Antihistamine (Cetirizine) orally
4) Antihistamine (Chlorphenamine) orally
5) Immediately start first line treatment - Antihistamine (Chlorphenamine) IV
6) Call PICU
7) Immediately start first line treatment - Adrenaline IM 1:10 000
8) Immediately start first line treatment - Adrenaline IM 1:1000
9) Metoclopramide
10) Adrenaline IV 1:100 if other treatment is unsuccessful
11) Immediately start first line treatment - Activated Charcoal
12) Inhaled Steroids
13) Oral prednisolone, in addition to first line treatment
14) Prednisolone IM as a first line treatment
15) Valproates
16) Midodrine
17) None of the above

if none of the above or any additional medications, please provide your options:

____

For peer review only

Next page of the online questionnaire…

On further questioning when the boy had recovered, he reported that the Chinese meal contained egg fried rice, beef, bean shoots, sesame, cashew nuts and peanuts. He has previously sometimes complained of itchy throat after eating chocolate and he had eczema in infancy worsened when eating egg. However, he has recently had egg without any reaction.

What tests would you do?

a) Full blood count
b) ECG
c) Echocardiogram
d) Urinalysis
e) Serum tryptase
f) Chest X-ray
g) EEG
h) sIgE or skin prick test to Egg
i) sIgE or skin prick test to Peanut, Cashew and Sesame
j) sIgG test to multiple food allergens
k) MRI
l) Spirometry
m) None of the above

if none of the above, or any additional tests, please provide your options:

____

____

What advice and further treatment would you give to this patient

____

____

For peer review only

Checklist from the https://www.equator-network.org/reporting-guidelines/good-practice-in-the-conduct-and-reporting-of-survey-research/ as recommended.

When reporting survey research, it is essential that a number of key points are covered (though the length and depth of reporting will be dependent upon journal style). These key points are presented as a 'checklist' below:

1. Explain the purpose or aim of the research, with the explicit identification of the research question.	✓
2. Explain why the research was necessary and place the study in context, drawing upon previous work in relevant fields (the literature review).	✓
3. Describe in (proportionate) detail how the research was done.	✓
a. State the chosen research method or methods, and justify why this method was chosen.	✓
b. Describe the research tool. If an existing tool is used, briefly state its psychometric properties and provide references to the original development work. If a new tool is used, you should include an entire section describing the steps undertaken to develop and test the tool, including results of psychometric testing.	✓
c. Describe how the sample was selected and how data were collected, including:	✓
iv.How were potential subjects identified?	✓
v.How many and what type of attempts were made to contact subjects?	✓
vi.Who approached potential subjects?	✓
vii.Where were potential subjects approached?	✓
viii.How was informed consent obtained?	✓
ix.How many agreed to participate?	✓
x.How did those who agreed differ from those who did not agree?<br><br>It is very hard to meet this recommendation due to a study design. Surveyed physicians voluntarily accessed the survey online and data available provides information and details only on those respondents who accessed the survey. We do not know how they differ from those physicians deciding not to undertake the survey.	✗
xi.What was the response rate?	✓
4. Describe and justify the methods and tests used for data analysis.	✓
5. Present the results of the research. The results section should be clear, factual, and concise.	✓
6. Interpret and discuss the findings. This 'discussion' section should not simply reiterate results; it should provide the author's critical reflection upon both the results and the processes of data collection. The discussion should assess how well the study met the research question, should describe the	✓

For peer review only - http://bmjopen.bmj.com/site/about/guidelines.xhtml

problems encountered in the research, and should honestly judge the limitations of the work.	
7. Present conclusions and recommendations.	✓

For peer review only
